# Selectivity on-target of bromodomain chemical probes by structure-guided medicinal chemistry and chemical biology

**DOI:** 10.4155/fmc-2016-0059

**Published:** 2016-05-19

**Authors:** Carles Galdeano, Alessio Ciulli

**Affiliations:** 1 Division of Biological Chemistry & Drug Discovery, School of Life Sciences, University of Dundee, James Black Centre, Dow Street, Dundee, DD1 5EH, UK; 2 Institut de Biomedicina de la Universitat de Barcelona (IBUB) & Departament de Fisicoquímica, Facultat de Farmàcia, Universitat de Barcelona, Av. Joan XXIII s/n, 08028 Barcelona, Spain

**Keywords:** BET bromodomains, chemical genetics, chemical probes, selectivity, structure-based drug discovery

## Abstract

Targeting epigenetic proteins is a rapidly growing area for medicinal chemistry and drug discovery. Recent years have seen an explosion of interest in developing small molecules binding to bromodomains, the readers of acetyl-lysine modifications. A plethora of co-crystal structures has motivated focused fragment-based design and optimization programs within both industry and academia. These efforts have yielded several compounds entering the clinic, and many more are increasingly being used as chemical probes to interrogate bromodomain biology. High selectivity of chemical probes is necessary to ensure biological activity is due to an on-target effect. Here, we review the state-of-the-art of bromodomain-targeting compounds, focusing on the structural basis for their on-target selectivity or lack thereof. We also highlight chemical biology approaches to enhance on-target selectivity.

Many physiological and pathological cellular processes are regulated by epigenetic mechanisms as a response to environmental stimuli [1]. It is well known that epigenetic regulation is controlled by chemical modifications on DNA and on post-translational modifications (PTMs) on histones, including acetylation, methylation and phosphorylation [2,3]. These chemical modifications on the histone amino acids are recognized by specific multidomain proteins able to write, read or erase them, and deregulation of these processes has been associated to disease [4–6]. Cancer was the first human disease linked to epigenetics. In 1983, Feinberg *et al.* found that genes of colorectal cancer cells were hypomethylated compared with normal tissues [7]. Since then, efforts have been devoted to increase knowledge in epigenetics and in particular to exploit understanding of these processes in order to develop new targeted molecular therapeutics [8,9].

Acetylation of the ε-amino group of lysine residues (KAc) is one of the most common modifications of histone tails [10,11]. Acetylation levels are reversibly maintained by histone acetyltransferases (HAT) and histone deacetylases (HDAC) that respectively write and erase this PTM [12]. HATs and HDACs are often deregulated in diseases through mechanisms that include aberrant expression levels, the occurrence of mutations as well as truncations, and chromosomal rearrangements [13]. From a drug discovery point of view, to date only a very limited number of HAT inhibitors have been described and the investigation of HAT inhibitors has been mostly limited to *in vitro* studies of growth inhibition of cell lines [14]. In contrast, several small molecules able to inhibit HDAC catalytic activity have been discovered and have also entered clinical trials with five examples already approved [15]. HDACs are to date the most explored epigenetic drug target family by the pharmaceutical industry [16].

In contrast, much less has been known of the reading process of acetylation marks in histones, and targeting protein interactions mediated by epigenetic readers of this PTM had remained largely unexplored until recently. However, this suddenly changed in 2010 with the publication of potent and selective triazolodiazepine-based inhibitors of Bromo and Extra-Terminal domain (BET) proteins, (+)-JQ1 and I-BET762 (Figure 1) [17,18], which were shown to have *in vivo* ontarget activity in models of NUT midline carcinoma and inflammation, respectively. BET inhibitors bound to highly conserved regions of BET proteins, called bromodomains, which were known to recognize the KAc modification in histones and other substrates.

These two groundbreaking discoveries demonstrated the high druggability of the bromodomain-KAc interaction and motivated further drug development efforts in this area. Since then, there has been an explosion of small molecules discovered or designed to target BET bromodomains as well as other bromodomains in the human phylogenetic tree. This has in part been facilitated by the high ‘ligandability’ toward fragment-like molecules, including small organic cosolvents such as DMSO and NMP [19]. This propensity to ligand binding has aided identification of high ligand efficiency hits from fragment screening campaigns that could be readily optimized for potency. In addition to providing leads that could be developed in drug discovery programs, these new molecules could be used to elucidate the biological function of bromodomains and their importance as therapeutic targets, in other words, as high-quality epigenetic chemical probes [20,21]. However, the high structural conservation of bromodomains pose a significant challenge toward developing suitable single-target selective inhibitors. Lack of selectivity poses a potential limitation to bromodomain inhibitors as chemical probes as it may confound the association of the cellular activity of a given probe to a particular bromodomain target. This has spurred mounting interest in developing more selective compounds.

Here we review recent advances to understand and exploit target selectivity of bromodomain chemical probes. We exemplify these efforts with case studies taken not only from the BET bromodomain subfamily but also from other bromodomain classes, as well as from studies identifying potential roles of bromodomains as off-targets, for example, of protein kinase inhibitors. We also highlight new developments in chemical biology approaches to enhance on-target selectivity of bromodomain probes and rationalize and alleviate off-target effects.

## Bromodomains & bromodomain-containing proteins: structure, inhibition by chemical probes & emerging role as drug targets

Bromodomains are an evolutionarily conserved family of ~110 amino acid modules found in histone acetyl transferases and other chromatin-associated proteins and transcriptional regulators [22]. The human genome encodes 46 of such bromodomain-containing proteins (BCPs) [23]. Each of the 46 proteins contains one to six bromodomains, giving a total number of 61 unique individual human bromodomain sequences. Based on sequence similarity, the whole human family of bromodomains can be divided into eight diverse subfamilies (group I–VIII) each containing at least three bromodomains and comprising proteins of diverse functions [24]. While most BCPs recognize KAc marks on histone tails, evidence suggests that bromodomains also are able to bind to acetylated proteins beyond histones [19]. An example of PTM recognition in nonhistone substrate is the HIV-1 Tat, which gets acetylated at position K50 and this allows it to associate to PCAF via recognition by the PCAF bromodomain [25]. Another example is in the p53 DNA damage response signal cascade, where acetylation of K382 on p53 enables the recruitment of transcriptional co-activator CREBBP via its bromodomain and ultimate activation of pro-apoptotic genes [26,27].

Despite their low overall sequence identity (~21%) bromodomains share a conserved fold comprising a bundle of four α-helices (named αZ, αA, αB and αC) linked by more diverse loop regions (Figure 2A). Two of these loops (ZA and BC) that make up the mouth of the KAc binding site show large sequence variations and contribute to substrate specificity [28,29]. Crystal structures with bound histone and substrate peptides have shown that KAc is recognized within a hydrophobic pocket and anchored by forming a conserved hydrogen bond between its side chain amide group and the side chain amide of a highly conserved Asn residue [30]. In addition, a water-mediated hydrogen bond is formed with the hydroxyl group of a conserved Tyr residue. Both residues are located at the bottom of the ZA loop of bromodomains (Figure 2B) [31]. Another characteristic feature at the base of the KAc recognition pocket is the presence of a network of water molecules that forms hydrogen bonds with the carbonyl group of the substrate KAc, forming the ZA channel (Figure 2B). Finally, in many bromodomains, including the BET subfamily members and a few others, a conserved stretch of three amino acids known as the ‘WPF shelf’ is found (Figure 2C). The presence or absence of the WPF shelf can be explored to achieve selectivity between BET and non-BET bromodomains [32].

Bromodomain-containing proteins (BCPs) are often deregulated in disease, and their bromodomains appear to have crucial roles to disease mechanism. Among the classes of BCPs that have been linked to disease are transcriptor co-regulators (e.g., BET protein BRD4 and ATAD2), transcriptional repressors (e.g., BAZ2A, also known as TIP5), chromatin-remodeling factors (e.g., BAZ1A, BPTF, CECR2), histone acetyltransferases (e.g., CREBBP and EP300) and E3 ubiquitin ligases (e.g., TRIM24) among others. Association of the role of many BCPs in disease has spurred the development of bromodomain inhibitors for drug discovery purposes [23]. Over the past 5 years, many inhibitors that target the BET subfamily of bromodomains have emerged. The BET subfamily, which comprises four members in humans (BRD2, BRD3, BRD4 and BRDT), take their name from the presence of two related tandem bromodomains named BD1 and BD2, able to specifically recognize different acetylation patterns in H3 and H4 histone tails [33]. Although BET bromodomains have a highly similar structural architecture, their function as individual domains is likely to be distinct. BET proteins are also likely to have distinct physiological roles, forming different protein– protein interactions (PPIs) and controlling specific regulatory networks [34]. For example, BRD4 acts as a transcription co-regulator [35,36]; BRD3 binds to acetylated GATA1 and regulates erythroid target genes [37]; while BRD2 is involved in cell-cycle progression [38], among other processes [39].

**Figure 1:**
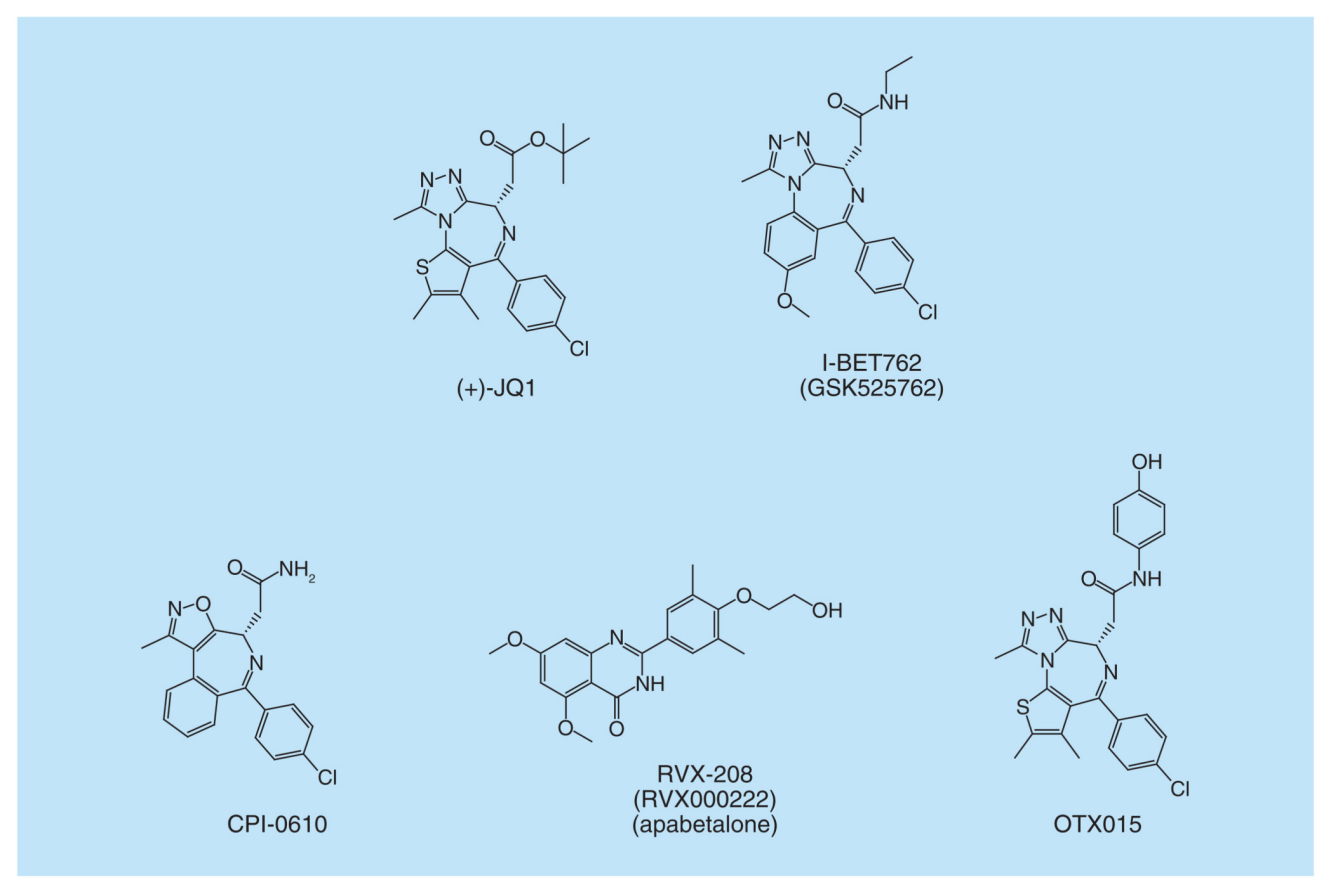
BET bromodomain inhibitors. Representation of the chemical structure of (+)-JQ1, I-BET762, CPI-0610, RVX-208 and OTX015.

To date, ten compounds blocking PPIs of BET bromodomains have entered clinical trials. One of them, RVX-208 (Figure 1, also named apabetalone and RVX000222) has reached Phase III clinical trials in autumn 2015 (NCT01728467), being the most advanced one. RVX-208, developed by Resverlogix Corp., has been evaluated in a total of seven clinical trials for the treatment of atherosclerosis and associated cardiovascular disease. In clinical Phase II, RVX-208 increased HDL-cholesterol and apolipoprotein A1 levels, as well as decreased the incidence of major adverse cardiac events (MACE). Additionally, reduction of MACE was accentuated in patients with diabetes mellitus. Three more BET bromodomains inhibitors have also reached Phase II clinical trials (OTX015, BMS-9861158 and GSK525762). OTX015 (Figure 1), developed by OncoEthix and Merck is involved in four different clinical trials for the treatment of acute leukemia and hematologic malignancies (NCT01713582), advanced solid tumors (NCT02259114), recurrent gliobastoma multiforme (NCT02296476) and in combination with azacitidine in patients in newly diagnosed acute myeloid leukemia that are not candidates for standard intensive induction therapy (NCT02303782). BMS-986158 (structure undisclosed) has been tested for multiple cancer indications alone and together with paclitaxel (NCT02419417). Finally, GSK525762, also known as I-BET762 (Figure 1) [18] is involved in two clinical trials: one to investigate safety, pharmacokinetics, pharmacodynamics and clinical activity in patients with NUT midline carcinoma and other cancers (NCT01587703) and a second one for patients with solid tumors with hematologic malignancies (NCT01943851). In addition to these four molecules, six other BET inhibitors have recently entered Phase I clinical trials and are being studied for both solid tumors and hematological malignancies: two compounds with a very similar structure to (+)-JQ1, TEN-010 [32] (NCT02308761, NCT01987362) and CPI-0610 [40] (Figure 1, NCT01949883, NCT02157636, NCT02158858); GS-5829 (structure undisclosed, NCT02392611, NCT02607228); BAY1238097 (structure undisclosed, NCT02369029); ABBV-075 (structure undisclosed, NCT02391480); and INCB054329 (structure undisclosed, NCT02431260).

Despite achieving several compounds in clinical trials, the development of BET inhibitors as chemical probes of individual BET proteins has remained a major challenge, due to lack of single target selectivity. Chemical probes are small molecules that elicit a cellular response by interacting with a specific protein inside the cell. In doing so, chemical probes can be used to explore the role of that particular protein in biological systems [41]. High-quality chemical probes must fulfill certain requirements that drugs do not necessarily do. Drugs must be safe and effective at treating disease, in contrast chemical probes must have a defined mechanism of action and should be as selective as possible, ideally for a single target [21,42]. The recent *Chemical Probes Portal* [43] provides information and guidelines on the criteria for establishing such high-quality chemical probes [21]. The web portal already contains several bromodomain inhibitors and could be an invaluable tool for the scientific community working in this field.

## The problem of chemical probe on-target selectivity in bromodomains

The high similarity within the different subfamilies of bromodomains and in particular at the level of their KAc binding sites poses inherent challenges for the development of bromodomain inhibitors as selective chemical probes. It is critical that a chemical probe molecule has a high degree of selectivity for one bromodomain over the other bromodomains to ensure that biological insights can be confidently ascribed to the function of the particular bromodomain of interest. Although for some subfamilies very high affinities have been described with compounds able to bind to bromodomains in the nanomolar range, achievement of exquisitely selective molecules for each member of the family has proven arduous. BET bromodomains are representative examples of this problem. Although dozens of potent compounds have been developed in the last few years, these are broadly pan-BET selective and none of them is able to bind in a selective way to a single member of the BET subfamily. In this context, several compounds with a poly-pharmacology profile for several bromodomains have been described, including a compound so-called bromosporine which showed reasonable binding affinity for different bromodomain family members [44]. Additionally, the availability of inhibitors with similar pharmacology but orthogonal chemotypes lends confidence to the interpretation of the biological activities of the different probes.

The selectivity criteria for chemical probes should be more stringent than for drug candidates. For drugs, safety is paramount and a level of promiscuity may even be beneficial to their efficacy profile and to minimize insurgency of drug resistance [21,45]. Guidelines on potency and selectivity of chemical probes should be considered as flexible and vary depending of the target family. For example, Cohen has described specific desirable criteria for kinase probes [46]. The Structural Genomics Consortium (SGC) has proposed the following criteria for a drug-like compound to be nominated as a chemical probe: IC_50_ or *K*_d_ <100 nM, selectivity >30-fold over proteins in the same family and significant cellular activity below 1 μM, matching quite well with the guidelines proposed by Workman *et al.* for high-quality selective chemical probes [47]. For other subfamilies outside of BET bromodomains, GlaxoSmithKline (GSK) has proposed that a minimum selectivity window of 2 and preferably 3 logs over BET bromodomains is required to ensure that an inhibitor has no cross-reactivity and that any biological response is not confounded by the strong BET-dependent response expected due to any BET off-target activity [48]. Large-scale *in vitro* selectivity profiling against other targets is also recommended, for example, against panels of kinases and GPCRs [49].

The typical strategy to gain selectivity in bromodomains has been to start drug discovery campaigns from fragments or anchoring scaffolds that bind to the KAc site [50], potentially across multiple bromodomains, and subsequently focus on optimizing the potency and selectivity for each individual bromodomains with substituents that interact with the less conserved sites at the edges of the KAc binding site. This strategy has been successful against several bromodomains, as described next.

## Selectivity for BETs versus non-BET bromodomains

Selectivity between different subfamilies of bromodomains has been achieved so far with a number of inhibitors. This has been aided by differences in the KAc binding sites, and by exploiting structural motifs that are specific for certain subfamilies. In fact, Vidler *et al.* [51] proposed a classification of human bromodomains based on the structural features that vary across the KAc binding sites, to complement that derived from sequence similarity of the entire domain [24]. In this new classification, eight clusters were defined by the presence of a unique signature of up to three amino acid residues in the binding site, shared by all members of each group. This classification should prove useful when determining selectivity of inhibitors and the potential to identify possibilities to transfer hit matter from one bromodomain to another. Although selectivity within members of a given group is also desired, this section will focus on selectivity across different groups with special emphasis on selectivity between BET bromodomains and the other subfamilies.

### BET bromodomains

**Figure 2:**
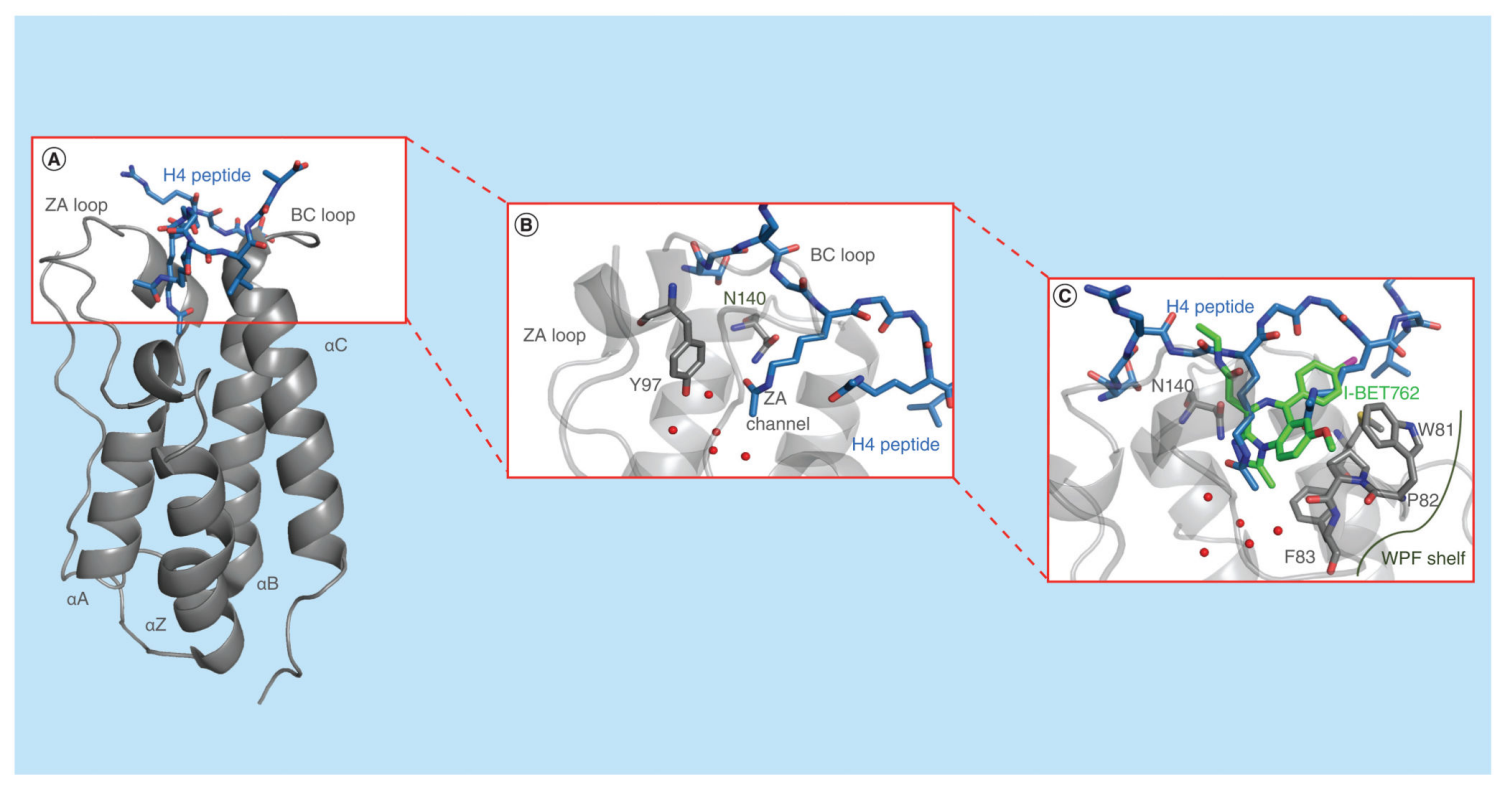
Structure and molecular recognition of BET bromodomains. **(A)** X-ray structure of the di-acetylated H4 peptide (double acetylation at H4K5acK8ac, in blue) bound to the BET bromodomain BRD4-BD1 (in gray, PDB 3UVW). **(B)** Highlighted the conserved Y97, N140 and the ZA channel of BRD4-BD1(PDB 3UVW). **(C)** Superposition of the di-acetylated H4 peptide (in blue, PDB 3UVW) and the I-BET762 inhibitor (in green, PBD 3P5O) bound to BRD4-BD1 (in gray), highlighting the residues forming the WPF shelf.

Since the pioneering publication of the two triazolodiazepine-based BET-selective inhibitors [17,18], various inhibitors of this subfamily have been developed, as extensively reviewed elsewhere [23,32,39,50,52,53]. BET bromodomains represent a highly druggable subfamily characterized by a long and accessible ZA channel and by the above-mentioned WPF shelf (Figure 2B & C). All BET bromodomains share this conserved motif, and filling the region of space adjacent to the WPF shelf with a small molecule has proven critical to achieve high binding affinities and to gain selectivity over non-BET bromodomains [54]. In general, BET inhibitors show a very high selectivity profile over members of other subfamilies. A few compounds have, however, exhibited off-target binding affinity for the CREBBP bromodomain comparable to that for BET bromodomains, for example, PFI-1 (Supplementary Figure 1) [55], I-BET726 (also named GSK1324726A) (Supplementary Figure 1) [56], XD14 (Supplementary Figure 1) [57,58], the 3,5-dimethylisoxazole derivatives (Supplementary Figure 1) [59,60] and a recent γ-carboline-based chemical series described by Ran *et al.* (Supplementary Figure 1) [61]. Additionally, anovelseriesof 3,5-dimethylisoxazole derivates (Supplementary Figure 1) compounds described by McKeown *et al.* demonstrated similar potency for BRD4-BD1 than for the first bromodomain of TAF1 [62].

### CREBBP/EP300 bromodomains

Achieving selectivity between CREBBP/EP300 and the BET subfamily has to date proven difficult as these bromodomains all possess long ZA loops bearing similar residues facing the KAc binding site. However, a few residues differ between the CREBBP and the BET bromodomains binding sites [60]. First, W81 from the top of the WPF shelf of BET bromodomains corresponds to a Leu in the CREBBP, making this part of the binding site of CREBBP more suitable in principle to accommodate bulkier ligands. Another residue that is considered to be key for attaining selectivity toward CREBBP is R1173, located at the entrance of the binding site of this bromodomain. This residue corresponds to D145 in BRD4-BD1. Some selectivity for the CREBBP bromodomain has resulted in part from exploiting specific cation–π interactions from ligands to the R1173 side chain [63–65].

**Figure 3:**
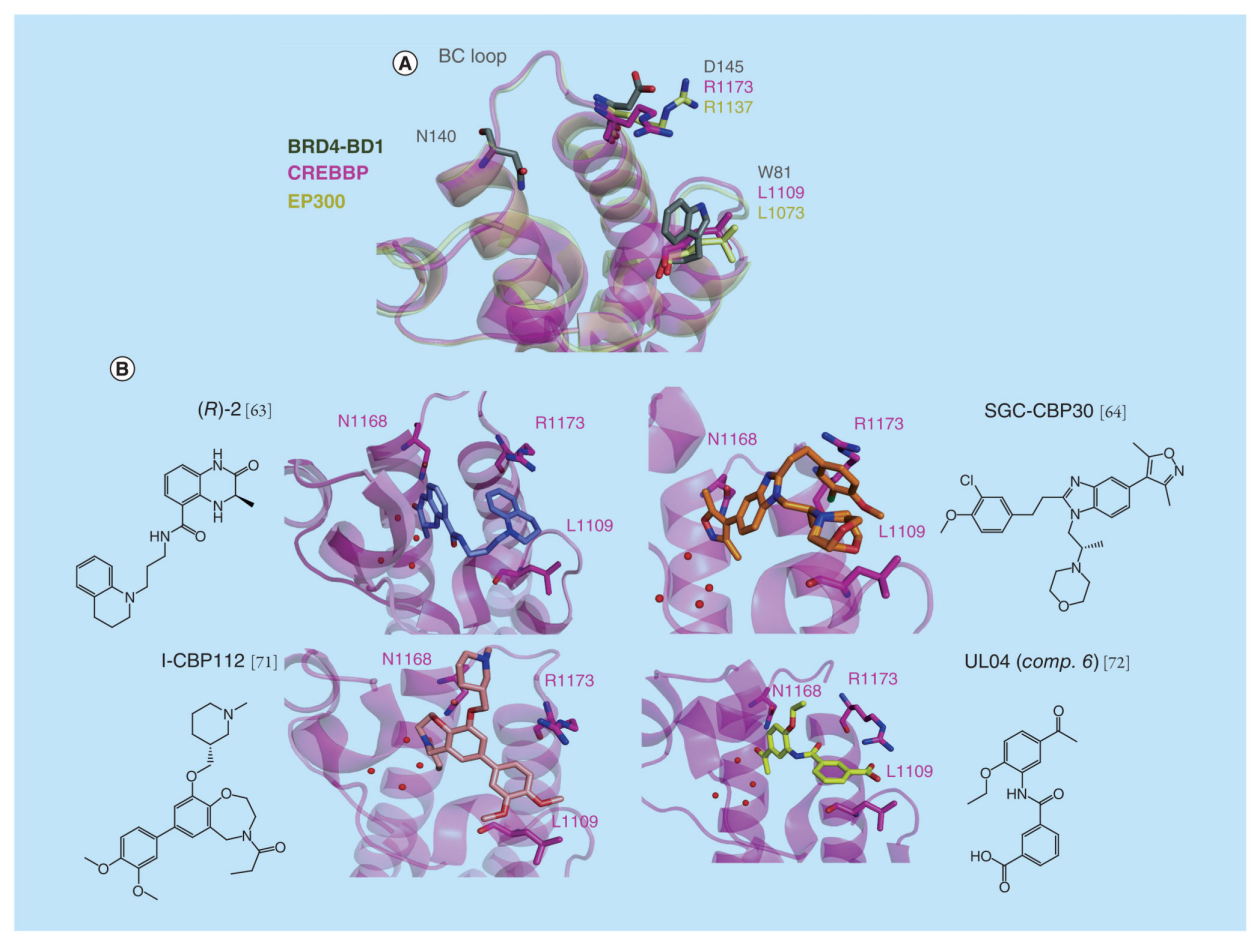
CREBBP/EP300 bromodomain. **(A)** Superposition of the x-ray structure of CREBBP (in pink, PDB 4NYW), BRD4-BD1 (in gray, PDB 3UVW) and EP300 (in yellow, PDB 5BT3). **(B)** X-ray structure of the ligands (*R*)-2 (PDB 4NYW), SGC-CBP30 (PDB 4NR7), I-CBP112 (PDB 4NR6) and UL04 (PDB 4TQN) bound to CREBBP (in pink). Note that SGC-CBP30 depicts two orientations of the morpholine group in the PDB 4NR7.

Several CREBBP inhibitors have been described in recent years, however, in most cases it has proven difficult to achieve strong selectivity over BET bromodomains [44,66–69]. The first series of potent inhibitors of CREBBP was described by Rooney *et al.* [63]. The best inhibitor reported, (*R*)-**2** (*K*_d_ = 390 nM by Isothermal Titration Calorimetry [ITC], Figure 3B) showed good selectivity over a few selected bromodomains but only modest selectivity over BRD4-BD1 (3.6-fold) comparable to that of compound ischemin (Supplementary Figure 1) developed previously [67]. In both cases, x-ray crystal structures (PDB 4NYW for (*R*)-**2** and 2L84 for ischemin) revealed the ability of these compounds to form interactions with the guanidinium group of R1173 in the BC loop of CREBBP (Figure 3B).

Hay *et al.* described SGC-CBP30 (Figure 3B), a highly potent (*K*_d_ = 21 nM by ITC) 3,5-dimethylisoxazole-based inhibitor for CREBBP bromodomain. SGC-CBP30 displays 40-fold selectivity over BRD4-BD1 and high selectivity over the other BET bromodomains apart from EP300 (*K*_d_ = 38 nM by ITC) [64,70]. The x-ray structure of SGC-CBP30 complexes to CREBBP (PDB 4NR7) highlighted the presence of a cation–π interaction between the guanidino group of R1173 and the aryl ring of the inhibitor, after a structural reorganization induced by the inhibitor (Figure 3B). More recently, the same group disclosed an x-ray structure with the benzoaxazepine-based inhibitor I-CBP112 (Figure 3B) co-crystallized with the CREBBP bromodomain (PDB 4NR6) [70]. I-CBP112 showed *K*_d_/IC_50_ of 170 nM by ITC and Biolayer Interferometry (BLI) respectively for the CREBBP bromodomain and a *K*_d_ of 625 nM for the EP300 bromodomain. Importantly, no interaction was detected by BLI for BDR4-BD1 and BRD2-BD2. Again, the aryl group of I-CBP112 interacts with R1173 (Figure 3B). For these two last series of selective compounds, the ability to engage in strong interaction with R1173 using electron-donating groups in the inhibitor aryl ring proved crucial [71].

The importance of R1173 as a key residue to gain selectivity for CREBBP against BET bromodomains was reinforced by a new series of ligands discovered by an *in silico* approach, with *K*_d_s down to nanomolar range for CREBBP bromodomain [72,73]. Selectivity factors of >65, 59 and 48 (compounds 6, 19 and 21, respectively in the original paper, Figure 3B & Supplementary Figure 1) were determined as BRD4-BD1-*K*_d_/CREBBP-*K*_d_ ratio by competition binding [72]. Finite-difference Poisson calculations on the minimized x-ray structure of compound 6 (in the original paper [72], also named UL04, Figure 3B) bound to the CREBBP bromodomain (PDB 4TQN) showed that half of the total electrostatic interaction energy is originated by the interaction between the carboxylic acid of the compound and the R1173 of CREBBP (Figure 3B). Although high selectivity over BET bromodomains was achieved, selectivity between CREBBP and EP300 bromodomains is yet to be described, and could prove far-fetched given the very high sequence identify and binding site similarity between these two domains (Figure 3A).

### BRD7 & BRD9 bromodomains

**Figure 4:**
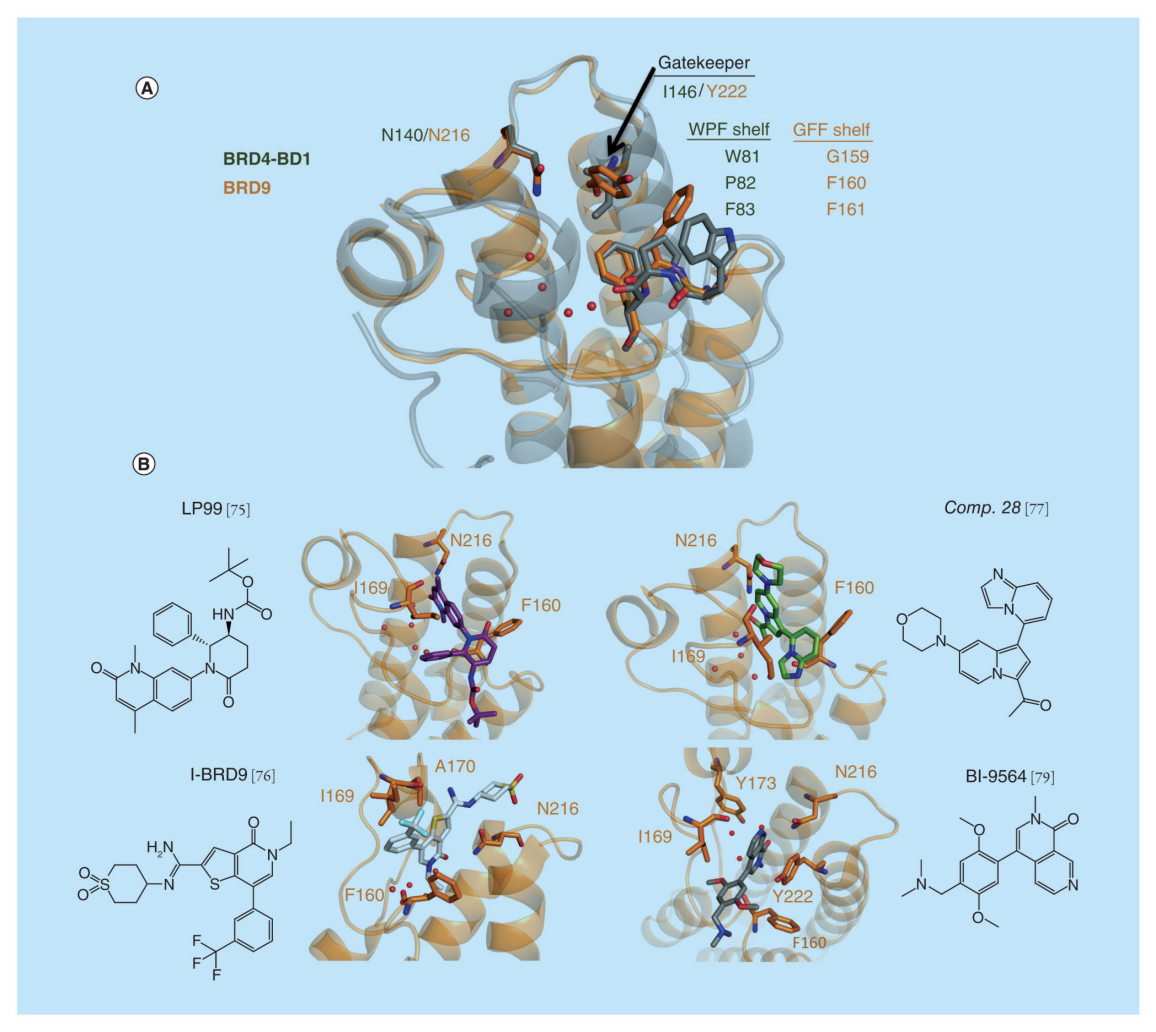
BRD9 bromodomain. **(A)** Superposition of the x-ray structure of BRD9 (in orange, PDB 4Z6I) and BRD4-BD1 (in gray, PDB 3UVW). **(B)** X-ray structure of the ligands LP99 (PDB 4X6I), compound 28 (PDB 5E9V), I-BRD9 (PDB 4UIW) and BI-9564 (PDB 5F1H) bound to BRD9 (in orange).

The elusive biological functions of BRD7 and BRD9 have motivated the development of selective chemical probes against the bromodomains of these proteins. The crystal structure of BRD9 revealed a different architecture of the ZA channel compared with BRD4-BD1. This region is much larger in BRD9, with residues A162, F163, P164, T166 and I169 forming a large hydrophobic cavity. Additionally, the replacement of the so-called gatekeeper residue I146 in BRD4-BD1 with Y222 in BRD9 results in blocking the access to the ‘GFF’ hydrophobic region that corresponds to the WPF shelf present in BET bromodomains, providing important structural differences that were deemed exploitable for ligand design (Figure 4A) [74].

Several BRD9 inhibitors have been reported. To date four distinct chemical series of compounds have been described that show selectivity toward BRD7 and BRD9 [75–79]. Starting from a verolactam fragment, Clark *et al.* aimed to extend interactions to the different hydrophobic regions between F160 and I169 of BRD9. This approach ultimately yielded LP99 (Figure 4B), a potent and selective inhibitor of BRD7 and BRD9, with a *K*_d_ against BRD9 and BRD7 of 99 and 909 nM, respectively by ITC [75]. LP99 was broadly profiled for selectivity by Differential Scanning Fluorimetry (DSF) against all expressible bromodomains (48 of 61 in the human genome), showing <1°C stabilization with other bromodomains [75]. Additionally, LP99 was found to be inactive versus 55 receptors and ion channels (CEREP panel) at 10 μM. The co-crystal structure of LP99 with BRD9 (PDB 4Z6I) confirmed that LP99 is stabilized by hydrophobic and aromatic residues in the KAc binding pocket and elucidated the structural basis for the stereospecific activity of (2*R*,3*S*)-LP99 (Figure 4B).

Another series of compounds that have shown selectivity for BRD9 and BRD7 were designed based on the BAZ2A/BAZ2B inhibitor GSK2801 (discussed later) [77,78]. The authors noted that the indolizine fragment of GSK2801 had also affinity for BRD9. After optimization of the starting fragment, compound 28 (from the original paper [77], Figure 4B) was found to be highly potent against BRD9 (*K*_d_ = 68 nM) and slightly less potent for BRD7 (*K*_d_ = 368 nM). The broader selectivity of this compound was assessed by DSF. Compound 28 is selective over most of the other bromodomain subfamilies, with modest affinity only for BRPF1B, CREBBP/p300 and FALZ. After determination of the x-ray structure of compound 28 bound to BRD9 (PDB 5E9V, Figure 4B), the selectivity observed could be rationalized by an interaction formed by the *C-1* imidazopyridine moiety with the hydrophobic region sandwiched between I169 and F160, which is much narrower in the BET bromodomains (Figure 4B) [77].

The high similarity between BRD7 and BRD9 bromodomains (around 80% sequence homology; 36% in terms of overall residues) [76,77] makes the development of a ligand selective only for one of these bromodomains a difficult task. However, this was achieved recently with the compound I-BRD9 (Figure 4B) [76]. I-BRD9 was identified through structure-based design, leading to greater than 700-fold selectivity over the BET subfamily members and 200-fold over the highly homologous BRD7 (*K*_d_ [BRD9, DiscoveRx] = 1.9 nM, *K*_d_[BRD7, DiscoveRx] = 380 nM, *K*_d_[BRD4-BD1, DiscoveRx] = 1400 nM) [76,79]. Several x-ray structures bound to BRD9 and BRD4-BD1 from several compounds during the extensive SAR process were solved in order to gain insights into the structural features that are responsible for the observed selectivity. Considering the basic nature of the amidine moiety of I-BRD9 (Figure 4B), it was proposed that when charged it would sit more favorably in the less hydrophobic environment alongside A170 of BRD9 than beside L94 of BRD4-BD1 (Figure 4B, PDB 4UIW). The additional selectivity of I-BRD9 for BRD9 over BRD7 is, however, difficult to rationalize since only an NMR structure of BRD7 (PDB 2I7K) is reported to date [80]. Potential contributions to the observed selectivity arise from differences in the constituent amino acids and in the architecture of the GFF region of BRD9 (G159 in BRD9 corresponds to A154 in BRD7) and the ZA loop (A162 in BRD9 corresponds to S157 in BRD7) of these two bromodomains.

Very recently, two inhibitors of the BRD9 and BRD7 bromodomains were developed using fragment-based screening and extensive structure-guided optimization by scientists at Boehringer Ingelheim in collaboration with SGC-Oxford and Cold Spring Harbor Laboratory (BI-7273 and BI-9564) [79]. The x-ray structure of BI-9564 bound to BRD9 (PDB 5F1H, Figure 4B) shows that the ligand makes water–bridged interactions with the specific BRD9 gatekeeper residue Y222, while keeping a double hydrogen bond with N216 and a water-mediated hydrogen bond with Y173. The compounds are potent (*K*_d_ in the 10^-9^–10^-8^ M range), highly selective (>1000-fold) against BRD4-BD1 and a panel of kinase and GPCR targets, have acceptable ADME and PK profiles, block AML cancer cell proliferation at ~1 μM and display efficacious antitumor activity in a xenograft model of human AML [79], providing the most high-quality chemical probes for BRD7/9 described to date.

Other inhibitors of BRD7/9 bromodomains have also been reported. These compounds either show mixed bromodomain pharmacology [44] or high affinity toward members of the BET subfamily [81], which make them unsuitable as chemical probes. Interestingly, the 9H-purine-based inhibitors described by Picaud *et al.* (Supplementary Figure 1) [81], although of modest selectivity, were able to induce an unprecedented rearrangement in the KAc recognition site (PDB 4XY8), which was not observed in the crystal structure of the same compound bound to BRD4-BD1 (PDB 4XY9). Specifically, the side chain of F77 (BRD9) rotated 120°, thus blocking the ZA-channel of the protein resulting in a steric clash around the ligands. These conformational changes could be exploited in principle to achieve selectivity for BRD7/9.

### BAZ bromodomains

**Figure 5:**
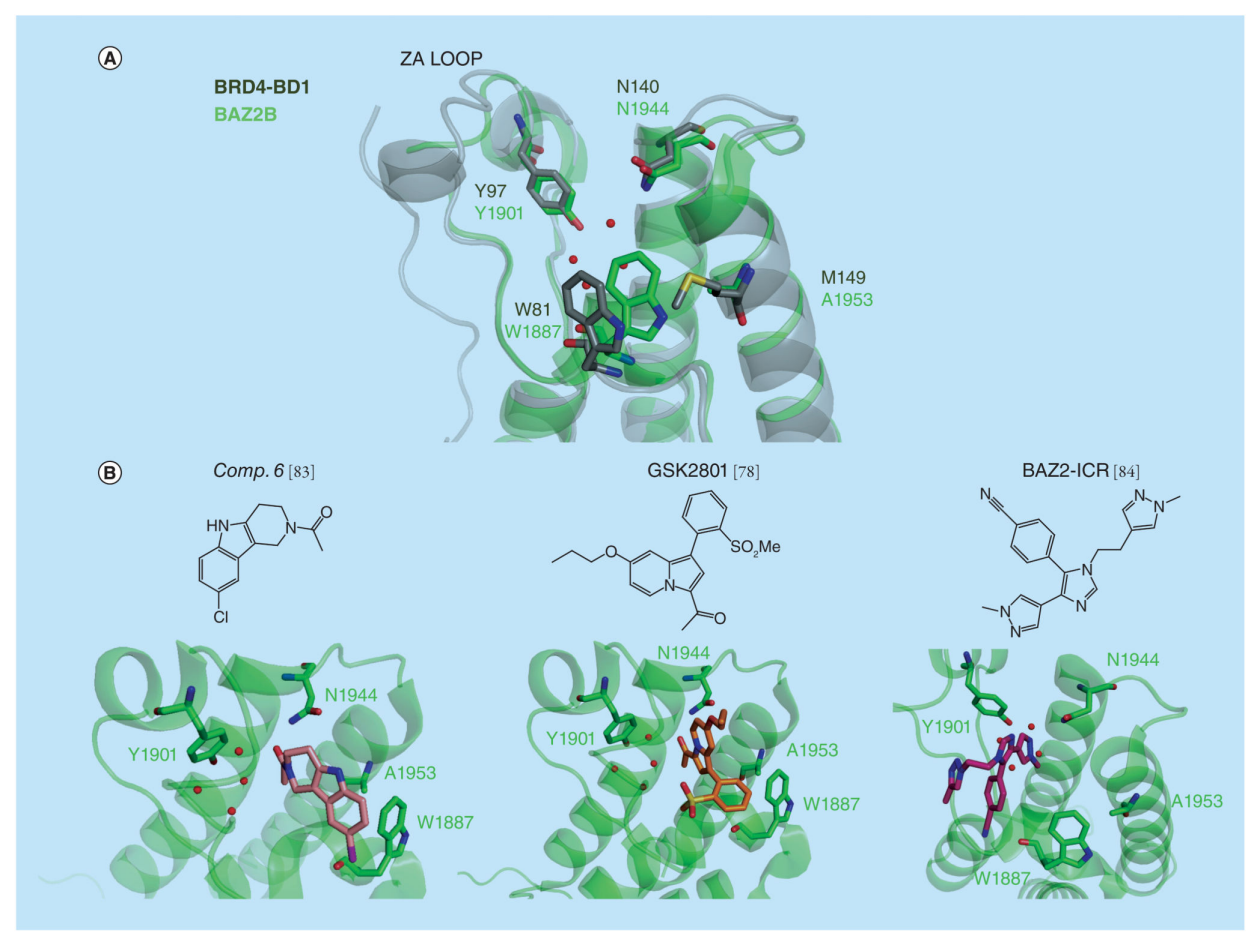
BAZ2B bromodomain. **(A)** Superposition of the x-ray structure of BAZ2B (in green, PDB 4NR9) and BRD4-BD1 (in gray, PDB 3UVW). **(B)** X-ray structure of the compound 6 (PDB 4NRA), GSK2801 (PDB 4RVR) and BAZ2-ICR (PDB 4XUB) bound to BAZ2B.

The BAZ subfamily of bromodomains (BAZ2A and BAZ2B) [82] had been considered one of the least druggable in the human genome [51]. They are characterized by a shorter ZA loop, making the pocket fairly open and reducing its druggability, and by lack of the ZA channel. On the other hand, the residue corresponding to M149 of BRD4-BD1 is much smaller in BAZ2B (A1953), and as a result cannot restrict the movement of the key Trp in the WPF shelf, potentially providing additional flexibility (Figure 5A).

In 2013, the first series of fragments with high micromolar affinity for the BAZ2B bromodomain were reported, which led to the structure-guided optimization of a γ-carboline-based inhibitor with single-digit micromolar affinity (PDB code of compound 6: 4NRA, Figure 5B) [83]. Fragment screening was also pursued by GSK and led to the development of the first nanomolar affinity compound, GSK2801 (Figure 5B), against the protein [78]. An indolizine fragment containing an acyl group mimicking the KAc recognition interaction was grown and optimized into GSK2801 based on structural information. A cocrystal structure (PDB 4RVR) showed that W1887 in BAZ2B is able to accommodate favorable π–stacking interactions with the inhibitor (Figure 5B). Bulky substituents in this part of the ligand introduced steric clashes against BET bromodomains, increasing selectivity as a result. GSK2801 showed *K*_d_s of 140 and 260 nM by ITC against BAZ2B and BAZ2A bromodomains, respectively. Only BRD9 and TAF1(L) bromodomains were detected as the major off-targets, after assessment of the GSK2801 selectivity for all human bromodomains by two orthogonal biophysical techniques, DSF and BLI.

In a parallel effort, Drouin *et al.* described BAZ2-ICR (Figure 5B), an inhibitor that targets BAZ2A and BAZ2B in the nanomolar range achieving highly selective binding over the other bromodomains, except for CERC2, in a DSF screen [84]. BAZ2-ICR displayed no off-target activity at 10 μM concentration against a panel of 55 receptors and ion channels. The co-crystal structure (PDB 4XUB) provided one of the first examples of a pyrazole moiety acting as a KAc mimetic efficiently filling out the binding pocket (Figure 5B) [84,85]. Noteworthy is the intramolecular π–stacking interactions formed by the compound in its bound conformation. This structural feature, often referred to as ‘hydrophobic collapse’, could help to achieve potency against other less druggable bromodomains, since it can provide shape complementary and extensive contacts in the case of more open and solvent-exposed binding sites, featuring less enclosed pockets, as is the case with BAZ2B.

### BRPF bromodomains

**Figure 6:**
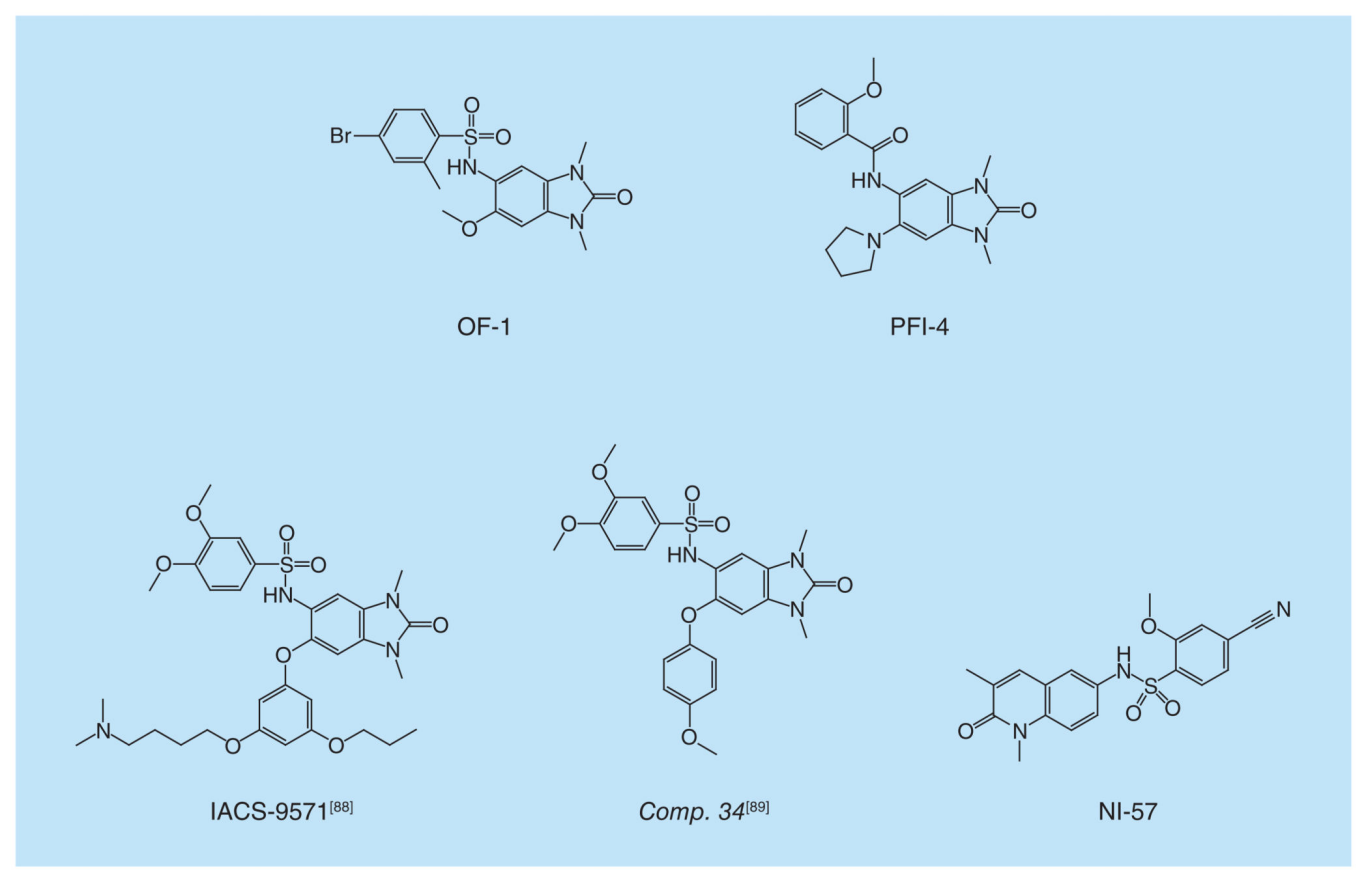
BRPF inhibitors. Chemical structure representation of OF-1, PFI-4, IACS-9571, compound 34 and NI-57.

**Figure 7:**
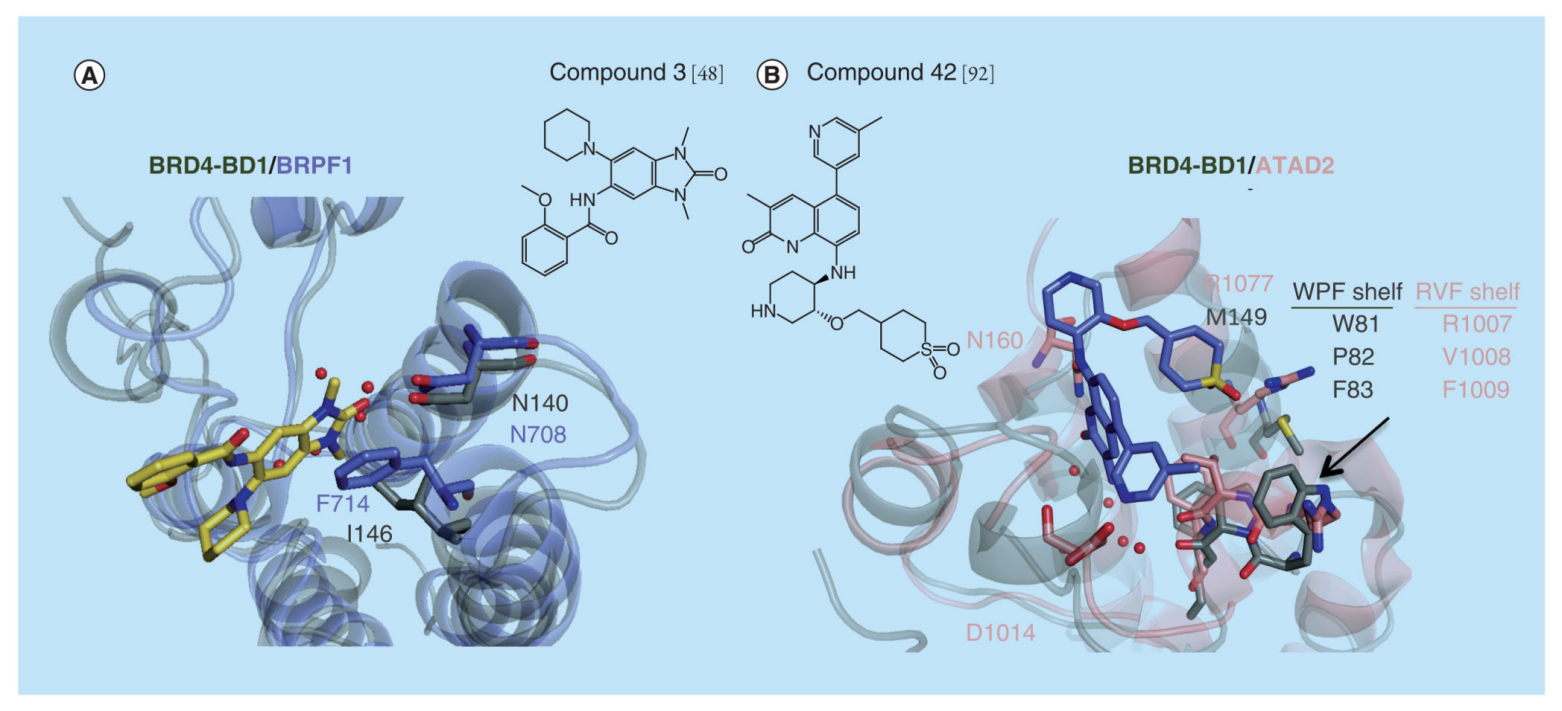
BRPF and ATAD2 bromodomains. **(A)** Superposition of the x-ray structures of compound 3 (in yellow) bound to BRPF1 (in purple, PDB 4UYE) and BRD4-BD1 (in gray, PDB 3UVW). **(B)** Superposition of the x-ray structures of compound 42 (in purple) bound to ATAD2 (in light pink, PDB 5A83) and BRD4-BD1 (in gray, PDB 3UVW).

The BRPF bromodomains KAc site closely resemble the BET and CREBBP bromodomains. This structural similarity translates in molecular recognition features, with identical fragments, for example, acetaminophen binding to BRPF bromodomains in a similar manner than for BET bromodomains and CREBBP [86]. One significant difference between BRD4 and BRPF1 is the hydrophobic ‘gatekeeper’ residue that forms one wall of the KAc site. This is I146 in BRD4-BD1 or V439 in BRD4-BD2 (Table 1). In contrast, a larger Phe residue occupies this position in the BRPF subfamily. This replacement promotes the selectivity seen in the 1,3-dimethyl benzimidazolones-based series described by Demont *et al.* (Figure 6 & Figure 7A, PDB 4UYE). The best in class of this series, compound 3 (in the original paper, Figure 7B) [48], showed a *p*IC_50_ for BRPF1 of 7.1, while the *p*IC_50_ for BRD4 was 4.3 [48]. Selectivity for BRPF1 over BRPF2 and BRPF3 (*p*IC_50_ of 5.1 and <4, respectively) could be achieved with compound 3, which was unexpected because the BRPF bromodomains themselves are highly conserved and share >65% sequence identity. This observation could be rationalized by the substitution of S592 in BRPF2 or N619 in BRPF3 with P658 in BRPF1. Compound 3 was tested in the BROMOscan panel of 35 bromodomains and showed high selectivity over other bromodomains [48].

Keeping the 1,3-dimethyl benzimidazolone core, scientists at the SGC reported OF-1 and PFI-4 (Figure 6). OF-1 (PDB 5FG4), developed in collaboration with Pfizer, showed good affinity to all of the members of the BRPF subfamily. Selectivity against other bromodomains proved >100-fold overall but the closest off-target effects were found against BRD4 (39-fold selectivity) and TIF1α (50% inhibition at 20 μM in an alphascreen assay). PFI-4 (PDB 5FG5, Figure 6) binds to BRPF1B with a *K*_d_ of 13 nM by ITC [87]. Selectivity data have not been published yet for PFI-4.

With the initial aim to obtain selective TRIM24 inhibitors, two molecules have been developed recently that are selective for BRPF and TRIM24, named IACS-9571 and compound 34 (Figure 6), respectively [88,89]. Selectivity over BRD4 was ensured by the structural similarity of the starting fragments to the ones used to develop BRPF inhibitors [48], however, this meant that no selectivity for TRIM24 over BRPF bromodomains could be obtained. A trend was seen in both cases; in that introduction of bulky groups at the 6-para-methoxy position of the 1,3-dimethyl benzimidazolone core scaffold increased dramatically the affinity toward TRIM24, while maintaining high affinity for BRPF.

Only one nonrelated 1,3-dimethyl benzimidazolone compound has been disclosed that is able to bind in a selective manner the BRPF bromodomains. NI-57 (Figure 6) [87] binds to the all BRPF subfamily members in the nanomolar range [*K*_d_s measured by ITC are 31 nM (BRPF1B), 108 nM (BRPF2) and 408 nM (BRPF2)]. NI-57 is highly selective against other bromodomains, including the BETs, as measured by both biophysical and biochemical methods. The closest off-target effect of NI-57 is against BRD9 (32-fold selective).

### ATAD2 bromodomain

Similar to BAZ2B, ATAD2 has been considered a poorly druggable bromodomain since the KAc binding site is significantly divergent with respect to those of other druggable bromodomains [51]. Although ATAD2 shares with most bromodomains the conserved residues responsible for KAc recognition, the KAc binding site is more open, polar and flexible than for example the one of BET bromodomains. When compared with BRD4-BD1, only three of seven residues lining the KAc binding pocket are shared, and the ZA loop is also two residues shorter than in BRD4. Additionally, W81 from the WPF is replaced by R1007, while M149 is replaced by R1077. In ATAD2, the region corresponding to the WPF shelf has been named ‘RVF shelf’ (R1007-V1008-F1009) (Figure 7B). Several screening campaigns have been performed to find fragment-size hits for ATAD2 [90,91], which yielded a number of weak-binding compounds. In parallel efforts, after a long and careful structure-based optimization a group at GSK developed potent and selective naphthyridone-based ATAD2 inhibitors [92,93]. In a first step, the authors were able to find low micromolar inhibitors for ATAD2 that, however, did not exhibit selectivity over BRD4 [93]. Subsequently, strategies to increase affinity and selectivity over BRD4-BD1 were explored. First, complementarity with the RVF shelf was optimized with appropriate polar substitutions in the compounds. Second, additional interactions with the backbone NH group of D1014 in the ZA loop were introduced, yielding the best compound of this series, compound 38 (in the original paper, Supplementary Figure 1) [92], with double-digit nanomolar binding affinities for ATAD2 (pIC_50_ATAD2 = 6.9 by TR-FRET) and showing >100-fold selectivity over the BET bromodomains. The x-ray structure of a close derivative, compound 42, bound to ATAD2 bromodomain was elucidated (PDB code of compound 42: 5A83, Figure 7B). However, the high potency and selectivity of compound 38 came at the expense of greater hydrophilicity that was solved in part with compound 46 (pIC_50_ATAD2 = 6.5 by TR-FRET, Supplementary Figure 1). Selectivity of compound 46 over the BET bromodomains was confirmed by Bromoscan with a window of at least >400-fold (*p*Ki by BROMOscan 7.7 against ATAD2 and 5.1 against BRD4-BD1). For the other bromodomains tested, the highest activity was with the second bromodomains of TAF1 and TAF1L (*p*Ki 7.3 and 6.9, respectively, by BROMOscan). In addition, chemoproteomics pulldown experiments were performed and *K*_d_s for the off-target bromodomains were determined [92].

### Other bromodomains

Few other bromodomains have been selectively targeted until today. Following a new ^19^F-NMR dual screening method using fluorinated tryptophan resonances on two bromodomain-containing proteins, Urick *et al.* discovered the first selective BPTF inhibitor, called AU1 (Supplementary Figure 1), from a library of 229 selected small molecules screened against BRD4-BD1 and BPTF [94]. SGC and Pfizer have collaborated to develop PFI-3 (Supplementary Figure 1, PDB 5DKC [SMARCA2] and 5DKD [SMARCA4]), a selective chemical probe for SMARCA 2/4 (89 nM for SMARCA4 by ITC) and PB1(5) (48 nM by ITC) bromodomains [95]. No interaction was observed with other bromodomains by DSF, and there was no cross-reactivity in a kinase panel of 36 kinases [95]. Despite the achieved potency and pan-selectivity, target bromodomain inhibition by PFI-3 did not impart the expected antiproliferative phenotype in relevant cancer cell lines, in contrast to what was observed with target knockdown by RNAi [95]. In another collaborative project, SGC and Novartis disclosed the selective CECR2 inhibitor NVS-CERC2-1 (Supplementary Figure 1) [87]. With an affinity of two-digit nanomolar and no interaction with the rest of the bromodomains in a BRD panel (48 targets), NVS-CERC2-1 is the only example to date of a chemical probe for this subfamily of bromodomains.

## Selectivity within BET bromodomains: BD1 versus BD2

**Figure 8:**
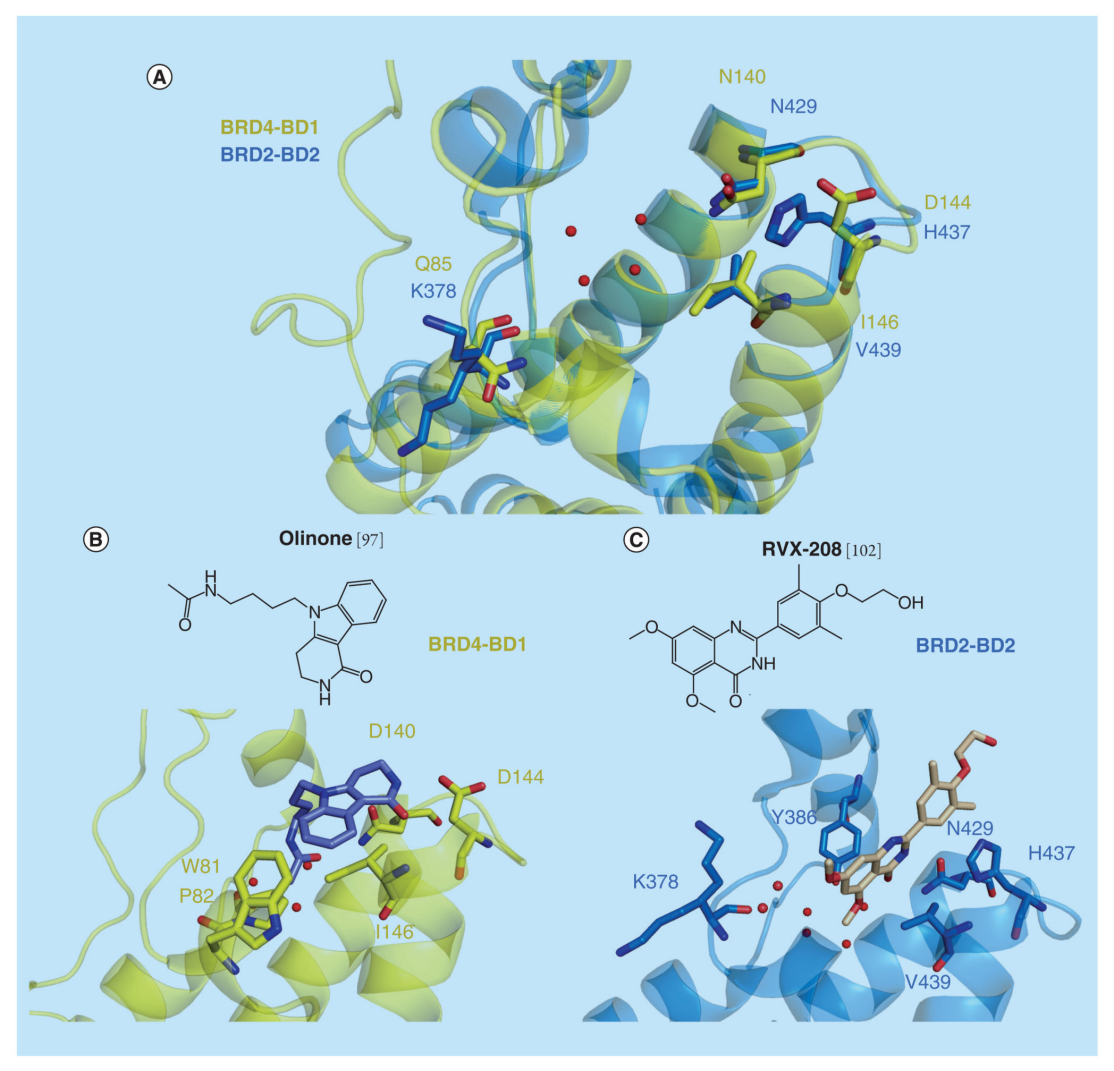
Selectivity between BD1 and BD2 BET bromodomains. **(A)** Superposition of the x-ray structure of BRD4-BD1 (in yellow, PDB 4QB3) and BRD2-BD2 (in blue, PDB 4J1P). **(B)** x-ray structure of olinone bound to the BRD4-BD1 (in yellow, PDB 4QB3). **(C)** x-ray structure of RVX-208 bound to the BRD2-BD2 (in yellow, PDB 4J1P).

All BET proteins are characterized by two N-terminal tandem domains (BD1 and BD2) followed by an extra terminal domain (ET domain). While BD1s and BD2s separately share greater than 75% sequence identity among themselves, only 38% cross-domain sequence identity is observed, suggesting distinct evolutionary ancestors and distinct functions [96]. However, all the BET bromodomains exhibit 95% sequence identity at the KAc binding pocket [97]. Sequence comparison between the two bromodomains of BRD4 in the proximity of the KAc peptide-binding site (Figure 8A & Table 1) shows three crucial residue positions that differ:

- Q85 in BD1 is a Lys residue in BD2 (K378);
- D144 in the BC loop of BD1 is a His residue in BD2 (H437); and
- I146 is a Val in BD2 (V439).

Although BET BD1 and BD2 show a high sequence similarity in the substrate-binding site, they exhibit different patterns of recognition of acetylated histone peptide target sequences [24]. A single BD1 or BD2 of BRD4 is individually able to interact with acetylated H4 peptide, however, only BD1 was found to recognize specifically N-terminal acetylated H4 peptides in a sequence-dependent manner, while BD2s were found to be more promiscuous [24]. As a general feature, BD1s of BET proteins appear to have a role in specifically recognizing H4 acetylation marks, whereas BD2s could have broader specificity toward acetylated substrates. The presence of two additional conserved regions, one containing the N-terminal cluster of casein kinase II phosphorylation sites (NPS) and one containing residues-enriched interaction domain (BID) situated downstream of BD2 in all BET proteins, would suggest that BD1 and BD2 may be differentially regulated by post-translational modification [98].

Several studies have shown that each BET bromodomain has a distinct function in the regulation of ordered gene transcription in chromatin possibly consequent to the interaction with lysine-acetylated histones or with other partner proteins. Gamsjaeger *et al.* showed that the two acetylated lysines in a sequence adjacent to the DNA-binding domain of the hematopoietic transcription factor GATA 1 are only recognized by the BD1 bromodomain of BRD3, while BD2 does not play any significant role in the recognition [37,99]. Shi *et al.* showed that Twist (an EMT-activating transcriptional factor) acetylated peptides could bind BRD4-BD2 with selectivity of >4-fold over BRD4-BD1 in a fluorescence polarization assay, suggesting that Twist preferentially interacts with the second bromodomain [100]. The preferential selectivity of the diacetylated Twist toward BD2 was attributed to a charged amino acid residue (D144 in BD1 or H437 in BD2) surrounding the KAc-binding pocket of BDs, together with additional residues beyond the diacetylation motif [100].

The same structural difference (D144 in BD1 or H437 in BD2) was highlighted as potentially playing a role in the observed selectivity of a compound called olinone for BRD4-BD1 over BD2 [97]. Olinone (Figure 8B) is a selective small-molecule inhibitor of the first bromodomain of BET proteins, consistently exhibiting over 100-fold higher binding affinity to BD1 (*K*_d_ = 3.4 μM) than BD2 (no detectable binding) by ITC for all of the BET bromodomains. A tetrahydropyrido indole chemical fragment (MS7972, Supplementary Figure 1) was identified as a hit from an NMR-based screen and showed modest activity as inhibitor of the CREBBP bromodomain [65]. Using MS7972 as a starting point, the authors incorporated longer acetamidoalkyl group substituents at the *N*-indole core, mimicking the natural KAc substrate. The x-ray structure of the BRD4-BD1/olinone complex (PDB 4QB3, Figure 8B) revealed that the alkyl group containing four methylene units adopts identical conformation and position as that of the acetylated-K5 side chain of the histone H4K5ac/K8ac peptide when bound to BRD4-BD1. Crucial for the BD1 selectivity, the modified triheterocyclic moiety containing a cyclic amide packs against the side chains of W81 and P82 and directly interacts with the specific BD1 residue D144 at the opening of the KAc binding pocket formed between the ZA and BC loops. In contrast, the corresponding residue in BD2, H437, would be predicted to clash with the cyclic moiety of olinone (Figure 8A).

The ability of olinone to bind preferentially the first bromodomain of BET proteins increases its potential to be used as a chemical probe to address how each of the paired bromodomains of BET proteins may function individually in the control of gene transcription in chromatin. In fact, comparison of the effect between olinone with MS417 (Supplementary Figure 1) that is equally potent against BD1 and BD2 showed dramatic differences in the biological response of oligodendrocyte progenitor lineage cells to compound treatments: whereas olinone promoted differentiation, MS417 inhibited it [97]. While these observations were interesting and potentially pointing to different roles for BD1 versus BD2, this was not conclusive due to the low potency of olinone and potential off-targets mediating its biological activity.

Preferential binding for BD1 over BD2 has been achieved with another class of diazobenzene-based BET inhibitors [101]. MS436 (Supplementary Figure 1), with an estimated *K*i of 30–50 nM for BRD4-BD1, and 10-fold selectivity over BD2, was the best inhibitor obtained after extensive lead optimization [101]. MS611 (Supplementary Figure 1) is another diazobenzene-based BET inhibitor with selectivity for BD1 against BD2 more recently described by the same group [97]. Surprisingly, MS611 showed 100-fold selectivity only for the BDs of the BRD4 bromodomain (*K*i = 0.41 μM [BRD4-BD1]; 41.3 μM [BRD4-BD2]), while no or very small differences in affinity were observed between the two BDs of BRD2 and BRD3. Further structural analysis is warranted to elucidate the basis of these remarkable differences observed between BET bromodomains.

While olinone represents the first selective BD1 inhibitor, RVX-208 (Figure 1 & Figure 8C) was the first BD2 selective inhibitor reported. RVX-208 (also named RVX000222 and apabetalone) was developed by Resverlogix Corp. and is now in clinical Phase III for the treatment of cardiovascular diseases associated with atherosclerosis and diabetes. RVX-208 is a small molecule that binds to BET bromodomains and competes for acetylated histone H4 peptide with a high preference for BD2 over BD1. Two papers published around the same time described the biophysical and structural characterization of this small molecule against BET bromodomains [102,103]. Although the results are very consistent along the two papers, some discrepancies about the absolute binding affinities measured by ITC of RVX-208 against BD1 and BD2 are seen. While Picaud *et al.* measured 23-fold selectivity for BRD2 [*K*_d_ = 5790 nM (BD1) and 251 nM (BD2)] and eightfold selectivity for BRD4 [*K*_d_ = 1142 nM (BD1) and 135 nM (BD2), McLure *et al.* measured 82-fold selectivity for BRD2 [*K*_d_ = 16900 nM (BD1) and 206 nM (BD2)] and 30-fold selectivity for BRD4 [*K*_d_ = 8930 nM (BD1) and 303 nM (BD2)]. These differences may reflect different conditions used in the experiments.

The high-resolution crystal structure of RVX-208 bound to BRD4-BD1 (PDB 4J3I and 4MR4) and BRD2-BD2 (PDB 4J1P and 4MR6) were resolved [102,103]. RVX-208 binds to the KAc pocket in a peptide-competitive manner, but unlike (+)-JQ1 it does not occupy the WPF region (Figure 8C). In all crystal structures, RVX-208 adopts a conserved binding mode. The carbonyl oxygen and the nitrogen atoms of the quinazoline ring system act as the KAc mimetic moiety, forming a hydrogen bond with the conserved N429 (BRD2-BD2) and a water-mediated hydrogen bond with Y386. The BD2 unique residue H433 in BRD2 flips into the KAc binding site packing against the phenyl ring of RVX-208, providing a possible explanation for the tighter affinity for BD2s (Figure 8C). Moreover, RVX-208 makes no direct interactions with residues unique to BD1, except to a water-mediated hydrogen bond with Q85 (K378 in BRD2-BD2).

Selectivity for the second over the first BET bromodomain using the IBET/(+)-JQ1 triazolodiazepine scaffold was observed by Baud *et al.* in the context of a bump-and-hole approach (see ‘Chemical biology & chemical genetics approaches to exploit selectivity’ section of this review) [104]. An analog of I-BET762/(+)-JQ1 (compound 28 in the original paper [104], Supplementary Figure 1) in which an indole group replaces the *p*-chlorophenyl group of I-BET762/(+)-JQ1 displayed a marked BD2 selectivity profile (20-fold). The observed selectivity was rationalized by observing in the co-crystal structures two distinct conformations (open and closed) for the His side chain (H433 in BRD2-BD2). The newly introduced indole group of the ligand exploits the closed conformation of H433 by forming an edge-to-face π-stack that would not be possible with the BD1 Asp residue (PDB 5DFD). These results indicate that BD2 versus BD1 isoform selectivity could be optimized with the aryl triazolodiazepine scaffold via comprehensive substitution of the parent *p*-chlorophenyl ring [104].

Finally, several compounds developed by Zenith Epigenetics (structure undisclosed) have also shown a remarkable selectivity for BRD4-BD2 toward BRD4-BD1. The best-in-class compound, ZEN297, showed a 60-fold selectivity (IC_50_ BRD4-BD1 = 1.2 μM; IC_50_ BRD4-BD2 = 0.02 μM) [105]. Despite their potential as chemical probes to elucidate the roles of the second bromodomains in BET proteins biology, the low potency of BD2-selective compounds for inhibiting tumor cell proliferation and *c*-MYC expression render them less attractive drug leads than BET inhibitors with BD1 activity.

## Selectivity within the BET subfamily

The development of an inhibitor selective for one of the bromodomain protein inside the BET subfamily has originated a tremendous interest in the academic and industrial settings. Although dozens of new molecular entities have been published and disclosed in the last 5 years, none of these have exhibited exquisite selectivity for a single BET protein over the others, and little is known about how to achieve this challenging selectivity. This is in part as a result of the high sequence identity within the KAc binding pocket of BET bromodomains (Table 1). However, very recently, Raux *et al.* indicated a different dynamic behavior of the ZA loop between the different BET bromodomains that could be exploited to fine tune selectivity [106]. In this work, a new class of xanthine-based inhibitors (Supplementary Figure 1) discovered using a ‘protein–protein interaction inhibition (2P2I)-oriented’ collection of compounds was able to yield the first described low micromolar selective inhibitor targeting BRD4-BD1 with a greater than tenfold ratio in binding affinity toward any other BET bromodomain. A potential hydrogen bond between the triazolo fragment of the new xanthine series and the BET conserved D88 residue that could be more stabilized in the case of BRD4-BD1, could in part explain the observed selective profiles. Additionally, van der Waals interaction with the side chain of the aforementioned Q85 in BRD4-BD1 (K378 in BRDs-BD2) contributed toward the preferential binding observed toward the BD1 bromodomain.

The pharmaceutical company Bayer disclosed in The Annual Meeting of The American Association for Cancer Research in 2015 the biochemical characteristics of BAY1238097 [107]. This compound, based on a novel scaffold (structure undisclosed), showed IC_50_ values of 63 nM for BRD4, 609 nM for BRD3 and 2430 nM for BRD2, corresponding to a selectivity of up to 39-fold for BRD4 versus BRD2. Chromatin immunoprecipitation (ChIP) experiments performed in MOLM-13 (AML) and MOLP-8 (MM) cell lines additionally revealed that BAY1238097 prevented binding of BRD4 to *c*-Myc regulatory region.

Another compound showing a degree of intra-BET selectivity *in vivo* is OTX015 (Figure 1), a BET inhibitor in clinical Phase III, which binds BET proteins with IC_50_ around 100 nM [108]. Coude *et al.* showed that OTX015 exposure decreased the expression of BRD2 and BRD4 and *c*-MYC, while BRD3 expression remained unaffected, in a broad range of acute leukemia cell lines and patient-derived leukemic samples [108].

## Chemical biology & chemical genetics approaches to exploit selectivity

### Bump-&-hole approach

The difficulty to obtain highly selective inhibitors for single BET bromodomains has motivated chemical biology/genetic approaches to address the challenges. Previous work on different enzymes, including protein kinases [109,110] provided proof-of-concept for generating allele-specific cofactors and inhibitors using a so-called ‘bump-and-hole’ approach. Using site-directed mutagenesis a ‘hole’ is introduced in the protein by replacing a natural amino acid with a smaller one, and this is compensated by introducing a bulky hydrophobic ‘bump’ into the natural ligand. This would be expected to confer high selectivity for the introduced mutant over the wild-type protein as the latter should not bind the ‘bumped’ ligand owing to steric hindrance.

**Figure 9:**
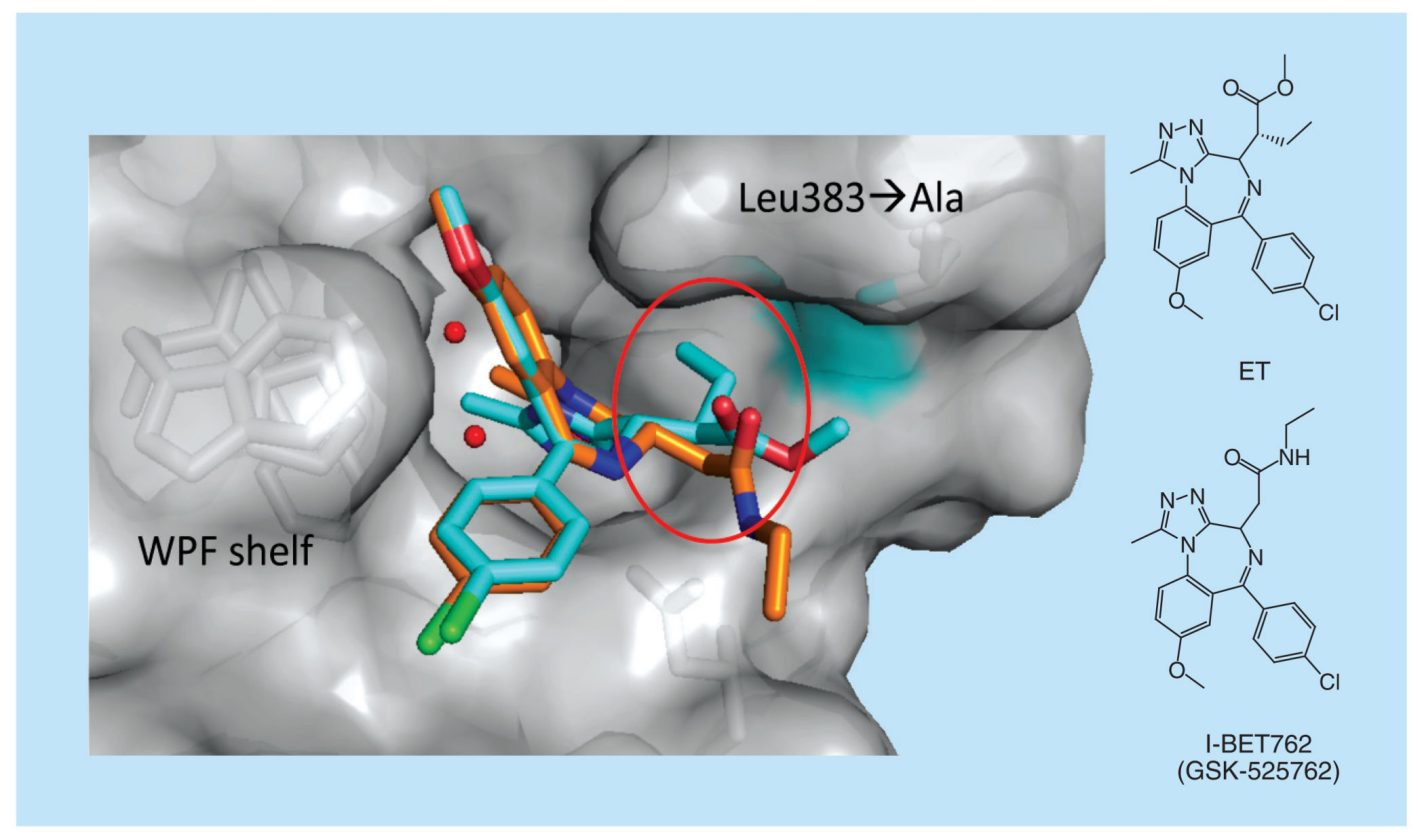
Bump-and-hole approach. Superposition of the x-ray structure of the ET (in cyan) bound to the BRD2-BD2 _L383A_ (in gray, PDB 4QEW) and parent ligand I-BET762 (in orange) bound to the BRD2-BD2 (not shown, PDB 5DFC).

Baud *et al.* successfully developed this approach to achieve exquisite single target selectivity of BET bromodomain chemical probes [111]. After sequence alignments and structural analysis guided by molecular docking, the authors identified a residue from the ZA-loop (L383 in BRD2-BD2) that is strictly conserved within BET bromodomains. Mutation of this into a smaller amino acid (Ala) created a ‘hole’ that did not significantly affect BET bromodomain stability and structural integrity, leading to mutant BET proteins of reasonable functionality that could accommodate a modified inhibitor. An ethylated derivate (named ET, Figure 9) of a methylester analog of I-BET762 was designed and found to bind to all mutant BET bromodomains with nanomolar affinities, while only binding the wild-type versions with single- to double-digit micromolar affinities. ET achieved up to 540-fold selectivity and no less than 30-fold (average 160-fold) across the entire BET subfamily, and high selectivity was retained within tandem constructs. To validate this strategy, x-ray structures of BRD2-BD2_L383A_ in complex with the methylated and ethylated bumped ligands were solved (PDB 4QEV and 4QEW respectively), showing that they adopt the same binding mode as I-BET762 and (+)-JQ1, positioning the respective methyl and ethyl substituents toward the hole introduced by the mutation (Figure 9). They went on and applied the approach in cells to demonstrate that selective pharmacological blockade of the first bromodomain of BRD4 is sufficient to displace the protein from chromatin, consistent with chromatin binding of BRD4 being principally influenced by BD1 over BD2 [111]. This study represents the first development of an allele-selective bump-and-hole approach against a protein–protein interaction target.

The same group attempted to exploit mutations in other conserved residues around the KAc binding site (V376A, W370F, W370H in BRD2-BD2) [104]. However, these new mutations did not lead to the desired increase in binding selectivity for mutant versus wild-type when targeted with appropriately designed bumped (+)-JQ1/I-BET762 analogs.

### PROTAC approach

With the aim to yield new chemical tools for studying BET bromodomain protein, Zengerle *et al.* described a new series of PROTACs (Proteolysis Targeting Chimera) molecules to trigger the intracellular destruction of BET proteins [112]. PROTACs are hetero–bifunctional small molecules that allow selective recruitment of a protein of interest to the ubiquitin-proteasome proteolytic machinery. By linking an optimized drug-like ligand able to bind to the von Hippel-Lindau E3 ligase protein to the BET bromodomain inhibitor (+)-JQ1, researchers at the University of Dundee were able to achieve rapid, effective and prolonged degradation of BET bromodomains with small molecules. Unexpectedly, the most potent novel PROTAC inhibitor, MZ1 (Supplementary Figure 1), showed a preferential degradation effect on BRD4 over the highly homologous BRD2 and BRD3 at relatively low concentrations. This selectivity window translated in a more BRD4-specific downstream transcriptional response in cancer cells treated with MZ1 as compared with treatment with (+)-JQ1 [112]. The VHL-targeting BET PROTACs reported in this study demonstrate for the first time the possibility for turning unselective or pan-selective inhibitors into chemical degraders of enhanced target selectivity profile. The authors speculated that the observed target selectivity could arise from preferential recruitment of BRD4 relative to BRD2/3 by MZ1 in a ternary complex with the VHL E3 ligase, or more efficient downstream polyubiquination of lysine residues on the surface of BRD4, which warrant further structural and mechanistic studies. Overall, MZ1 showed improvements over (+)-JQ1 as a chemical probe due to its more limited transcriptional response and as a potential molecular therapeutic, providing unique opportunity to validate the therapeutic benefit of selective BRD4 removal, for example, compared with or in addition to pan-selective BET inhibition.

A similar approach to degrade BET bromodomains with a PROTAC molecule was pursued by Lu *et al.* [113]. In this case, OTX015 was used as the BET bromodomain binding ligand, which was joined to a phtalimide binding ligand for the E3 ligase *cereblon*. Cereblon-targeting BET PROTAC ARV-825 (Supplementary Figure 1) induced effective degradation of all BET proteins and a strikingly more pronounced anti-proliferative and *c*-MYC suppressive effect compared with (+)-JQ1 and OTX015 alone [113,114]. Interestingly, no target degradation selectivity between BRD2, BRD3 and BRD4 was observed. A similar PROTAC strategy was followed by Winter *et al.* where (+)-JQ1 was conjugated to the same phtalimide ligand for cereblon, which induced cereblon-dependent BET protein degradation in cells and *in vivo* and delayed leukemia progression in mice [115]. Although the new compound dBET1 (Supplementary Figure 1) showed pan-selectivity for inducing degradation of BET proteins, no noteworthy intra-BET selectivity was observed [115].

### Chem-Seq approach

The ability to map direct interactions of molecules with chromatin genome-wide could provide important information on the on-target effect of small molecules, aiding biological insights. Anders *et al.* used *Chem-seq*, a method based on chemical affinity capture and parallel unbiased DNA sequencing, to investigate the genome-wide binding of the bromodomain inhibitor (+)-JQ1 to the BRD2, BRD3 and BRD4 in multiple myeloma cells (MM1.S) [116]. The authors showed that the genomic sites bound by a biotinylated derivate of (+)-JQ1 (bio-JQ1, Supplementary Figure 1) are highly similar to the sites occupied by the native BRD2, BRD3 and BRD4 proteins. However, inspection of gene tracks for regions differentially occupied by bio-JQ1 provided evidence that bio-JQ1 tends to cooccupy enhancers where there are substantial BRD4 signals, and lower signals for BRD2 and BRD3. These results indicated that the pattern of (+)-JQ1 occupancy of chromatin is most strongly correlated with that of BRD4 in MM1.S cells [116].

## Off-target effects

### Dual bromodomain/kinase inhibitors

The target selectivity and potential off-target effects of kinase inhibitors remain an important issue in their utilization as kinase chemical probes [117]. Several recent studies have revealed interesting off-target binding of BET bromodomains by diverse kinase inhibitors.

**Figure 10:**
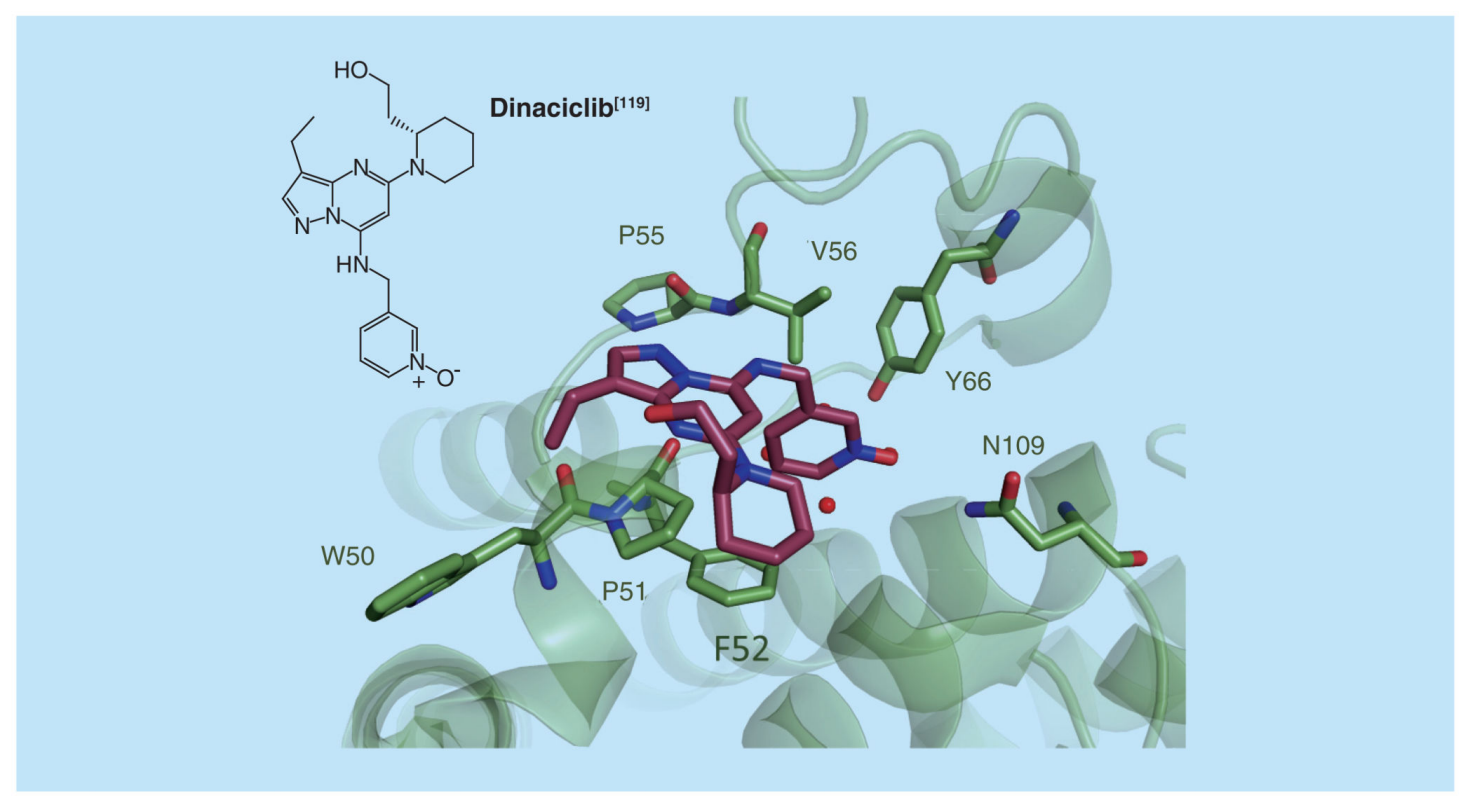
Dual bromodomain/kinase inhibitors. X-ray structure of the dinaciclib (in magenta) bound to the BRDT-BD1 BET bromodomain (in green, PDB 4KCX).

Intrigued by the observation that BRD4 exerts kinase activity against Pol II [118], Martin *et al.* decided to elucidate the x-ray structure of the potent CDK inhibitor dinaciclib with the BET bromodomain BRDT-BD1 (PDB 4KCX, Figure 10) [119]. Surprisingly, the structure revealed that dinaciclib binds exactly to the KAc binding site of BRDT, albeit with a different binding mode than (+)-JQ1, resulting in a suboptimal binding affinity compared with (+)-JQ1 (*K*_d_ for BRDT-BD1 of 37 μM in qPCR-based assay). The pyridine oxide moiety of dinaciclib acts as a KAc mimetic interacting with the crucial Asn amino acid (N109 in BRDT-BD1) and the pyrazolo-pyrimidine moiety lies parallel to the WPF shelf, making additional water-mediated hydrogen bond with the backbone of ZA channel residues P55 and V56. An additional profiling of dinaciclib against a panel of 24 bromodomains revealed that apart from BET bromodomains, the only other bromodomains with binding potential for dinaciclib were TAF1 and TAF1L. These findings were consistent with the hypothesis that BET proteins could be potential off-targets of ATP-site-directed kinase inhibitors. Because of the potential of synergistic pharmacology between kinase and BET inhibition in cancer cell lines, several kinase inhibitors have since been investigated for their potential to bind to BET bromodomains [49,120,121].

Through a robotic co-crystallization screening campaign Ember *et al.* demonstrated that a number of protein kinase inhibitors currently in the clinic, for example, BI-2536 (Supplementary Figure 1) and TG-101348 (Supplementary Figure 1), a PLK1 and a JAK2-FLT3 kinase inhibitor, respectively, also bind to members of the bromodomain family with a potency and selectivity that would be predicted to be therapeutically relevant [49]. A total of 14 kinase inhibitors were characterized by Ember *et al.* as BRD4-BD1 inhibitors, the majority of them showing micromolar inhibition of BET proteins. x-ray cocrystal structures showed that most of the kinase inhibitors take a slightly different conformation when they bind to the bromodomain compared with when they bind to the ATP site of the kinase, which may result in unfavorably high energy states, and consequently, reduced binding potential toward BET bromodomains. Importantly, none of the kinase inhibitors investigated in the paper interact with the characteristic water network of the KAc binding site, opening opportunities for the development of a novel class of BET bromodomains, directly interacting with the residues of the binding site. In parallel, Dittman *et al.* following a quantitative chemoproteomic approach demostrated that LY294002 (Supplementary Figure 1) and its negative control analog LY303511 (Supplementary Figure 1), two inhibitors of PI3K enzymes, were both able to block the first, but not the second, bromodomain of BRD2, BRD3 and BRD4 [120]. Further, it was found that the cellular and transcriptional effects of LY303511 were accounted for largely by their off-target interaction with BET bromodomains.

In a separate study, Ciceri *et al.* also reported that several clinical kinase inhibitors, such as BI-2536 and TG-101348 (Supplementary Figure 1) inhibit bromodomains with therapeutically relevant potencies [122]. In fact, PLK1 and its inhibitor BI-2536 has been the focus of some of the efforts to develop dual inhibitors [123]. These combination of activities on independent oncogenic pathways exemplify a new strategy for rational single-agent poly-pharmacological targeting and provide a new structural framework for the rational design of next-generation BET-selective and dual-activity BET-kinase inhibitors.

### GABA inhibitors

For many years, the benzodiazepine chemical group has been considered as a ‘privileged scaffold’ in drug discovery [104,124,125]. A large number of approved inhibitors of the GABA receptors contain the benzodiazepine moiety. In addition, several bromodomain inhibitors in clinical trials such as I-BET762, (+)-JQ1 and OTX15 (Figure 1) also have a benzodiazepine-based structure.

**Figure 11:**
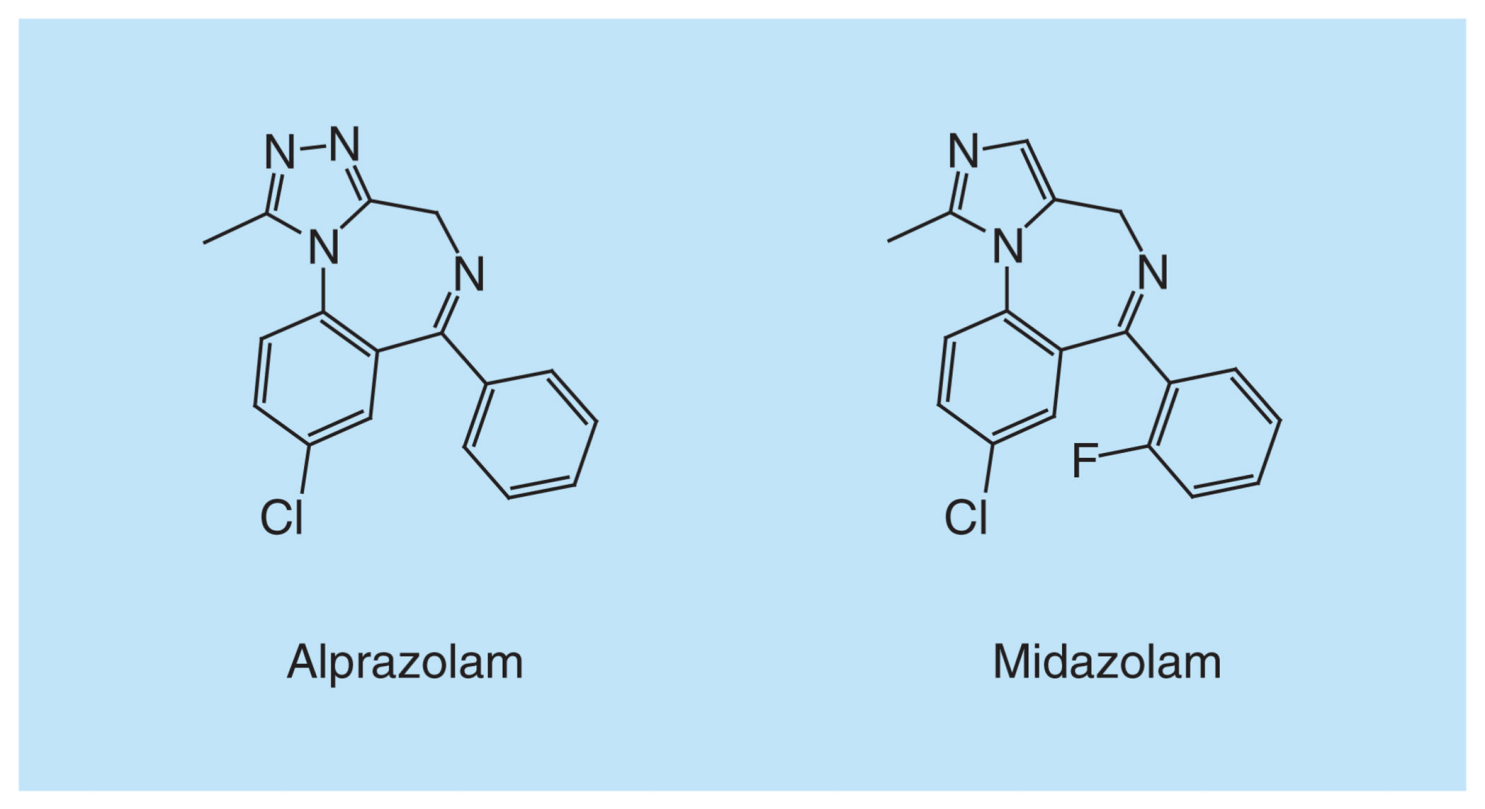
GABA inhibitors. Chemical structure representation of alprazolam and midazolam.

Filippakopoulos *et al.* provided insights into the structure–activity relationships and selectivity of the approved GABA inhibitors toward the BET bromodomains [126]. They found that alprazolam (Figure 11) binds with low micromolar affinity to BRD4-BD1. The co-crystal structure of alprazolam with BRD4-BD1 showed that the triazolo ring of alprazolam forms a hydrogen bond with the conserved Asn residue. Unexpectedly, also midazolam interacts with BET bromodomains, preferentially with BD2, in the micromolar range of affinity. Co-crystal structure of midazolam with BRD4-BD1 shows that although midazolam lacks the hydrogen bond forming nitrogen in the triazolo group, it is able to bind to this BET bromodomain after a reorganization of the network of conserved water molecules inside of the KAc binding site [126]. However, the micromolar affinities of these compounds make unlikely that this activity will cause side effects due to inhibition of BET bromodomains.

## Conclusion & future perspective

Epigenetic control of gene expression plays a significant role in a variety of diseases, including cancer and inflammation. Protein readers of histone acetylation and their bromodomains have emerged as attractive drug targets for these diseases, yet little is known about their individual biological function, how they work inside cells in both physiological and pathophysiological contexts, and how they should be best modulated pharmacologically. For these reasons, much interest has spurred in epigenetic medicinal chemistry and drug discovery, and specifically in the structure-guided development of chemical probes for bromodomains, to help fill these knowledge gaps and validate new potential drug targets.

In this review, we have highlighted the state-of-the-art in the field, focusing on the *in vitro* on-target selectivity of the probes reported to date, as measured using biophysical techniques mostly against purified proteins. Robust *in vitro* selectivity profiling of chemical probes is advocated, using appropriate assays such as the BromoScan assay provided by DiscoveRx and related assays over less quantitative methods, for example, DSF, as well as selectivity profiling against key target classes, for example, kinases and GPCRs. We expect efforts in future to focus on assessment of target engagement selectivity inside cells in an unbiased fashion. Progress in this direction will likely take advantage of new developments in molecular biology and genome editing, for example, by clustered regularly interspaced short palindromic repeats (CRISPR)-Cas9 [127], and the translation of biophysical measurements directly in cellular environment, as exemplified by cellular thermal shift assay (CETSA) [128], cellular thermal profiling by mass spectrometry [129] and methods using bioluminescence resonance energy transfer (BRET) such as NanoBRET assay [130,131].

The cutting-edge progress achieved in recent years in the field by both industrial and academic scientists demonstrates that it is possible to obtain some degree of target selectivity within selected bromodomain subfamilies. For chemical probes targeting non-BET family members, selectivity against BET bromodomains, particularly BRD4-BD1, is considered a critical requirement to eliminate any unwanted BRD4-dependent cellular effect. It has nevertheless remained a challenge to achieve intra-family single target selectivity. When this cannot be achieved by targeting the KAc pocket, alternative protein domains or more specific PPI binding sites outside the KAc pocket will likely need to be explored. It is also envisaged that covalent reversible or irreversible inhibition, which has so far not been much exploited against bromodomains, could help to enhance on-target selectivity.

In addition to conventional inhibitors, more sophisticated chemical biology approaches such as bump-and-hole and PROTACs have demonstrated that they can lead to single-target selectivity even when starting from the pan-selective inhibitor (+)-JQ1. Such new, more refined chemical tools are poised for many applications in the near future to dissect individual physiological roles of BET proteins *in vivo*, and could be widely extended to other BCPs as well as other epigenetic reader domains.

Despite the extraordinary progress to date, there still remain many challenges facing the field ahead. Bromodomains have proven to be highly ‘ligandable’ targets, especially for small-molecule fragments, and to be particularly suitable to biophysical fragment screening. The readiness with which nanomolar binding affinities can be achieved for a given bromodomain seems to be dependent on the nature of each target, yet inhibitor potency can be achieved with most bromodomains. From a drug discovery perspective, modulation of BET proteins with bromodomain inhibitors is now a validated therapeutic approach. It remains to be seen to what extent the many inhibitors for non-BET bromodomains that are available and will continue to emerge can exert a desired level of cellular efficacy, and consequently the question of which target will prove to be ‘druggable’ as well as ‘ligandable’ remains open. Validated drug targets will likely be determined more by the biology of individual BCPs, than by the ligand-ability of their bromodomain. Effective coupling of chemical with biological target validation approaches, surmounting inherent challenges associated with the complexity of many BCPs and their function, will be paramount to ensure the most relevant targets are prioritized in future. Nevertheless, we predict that new small molecule probes will continue to be developed in this area, providing yet more tools for the scientific community to use to help answer important biological questions.

## Supplementary data

To view the supplementary data that accompany this paper please visit the journal website at: www.future-science.com/doi/full/10.4155/fmc-2016-0059

Supplementary Data

## Figures and Tables

**Table 1:** Sequence similarity between residues of the KAc binding sites of BRD2-BD1, BRD3-BD1, BRD4-BD1 and BRD4-BD2

Motifs	BRD2-BD1	BRD3-BD1	BRD4-BD1	BRD4-BD2
Conserved Asn	N156	N116	N140	N433
Conserved Tyr	Y113	Y73	Y97	Y432
WPF motif	W97	W57	W81	W374
WPF motif	P98	P58	P82	P375
WPF motif	F99	F59	F83	F376
ZA loop	D104	D64	D88	D381
ZA loop	A105	A65	A89	V382†
ZA loop	V106†	I66†	V90†	E383†
ZA loop	K107	K67	K91	A384†
ZA loop	L108	L68	L92	L385
ZA loop	G109†	N69†	N93†	G386†
ZA loop	L110	L70	L94	L387
ZA channel	R100†	Y60	Q84†	Y377
ZA channel	Q101	Q61	Q85	K378†
ZA channel	P102	P62	P86	P379
ZA channel	V103	V63	V87	V380
Gatekeeper	I162	I122	I146	V439†
BC loop	T159	T119	G143†	D436†
BC loop	D160	D120	D144	H437†
BC loop	D161	D121	D145	E438†

† The residues that differ between the BET bromodomains and can be exploited to achieve selectivity.

## Executive summary

- With the publication in 2010 of BET inhibitors (+)-JQ1 and I-BET762, there has been an explosion of small molecules discovered or designed to target bromodomains in the human family.
- The high similarity within the different subfamilies of bromodomains, in particular at the level of their KAc binding sites, poses inherent challenges to the development of selective bromodomain inhibitors for delivering high-quality chemical probes.
- Despite achieving several compounds in clinical trials, the development of BET inhibitors as chemical probes of individual BET proteins has remained a major challenge, due to the lack of single target selectivity.
- We exemplify the efforts to obtain selective bromodomains-class inhibitors with case studies taken not only from the BET subfamily but also from bromodomain targets in other subfamilies.
- The ability of BET inhibitors to bind preferentially BD1 or BD2 of BET proteins increases their potential to be used as a chemical probe to address how each of the paired bromodomains of BET proteins may function individually in the control of gene transcription in chromatin.
- Innovative chemical biology and chemical genetic approaches such as bump-and-hole, PROTACs and Chem-Seq approaches allow to address and enhance on-target selectivity and efficacy, and interrogate context-dependent mode of action of BET inhibitors.
- Several studies have revealed interesting and unexpected off-target binding of BET bromodomains by inhibitors previously developed against other targets, such as protein kinases and receptors.
- We have described key challenges facing the development of bromodomain inhibitors as highly selective chemical probes, of the quality necessary for use as tools to address biological questions.
- Future efforts to attempt to solve these challenges should focus on development of innovative/alternative approaches including assessment of target engagement selectivity in bromodomains inside cells in an unbiased fashion; covalent reversible or irreversible inhibitor design; development and application of sophisticated chemical biology approaches to enhance chemical probing; and finally, closer synergy between chemical and biological target validation.
