# Supplementary material for: Selectivity on-target of bromodomain chemical probes by structure-guided medicinal chemistry and chemical biology
Source: Future Med Chem. Author manuscript; Available in PMC 2017 Feb 22. (PMC5321501; doi:10.4155/fmc-2016-0059)
Supplement: Supplementary Data [file NIHMS70267-supplement-Supplementary_Data.docx]

**SUPPLEMENTARY FIGURE 1**
